# Clonal Hematopoiesis Across the Cancer Treatment Continuum: A Scoping Review of Clonal Selection, Evolution, and Clinical Outcomes

**DOI:** 10.3390/cancers18203398

**Published:** 2026-10-21

**Authors:** Abdullah M. AlShahrani

**Affiliations:** Department of Basic Medical Science, College of Applied Medical Sciences, King Khalid University (KKU), Abha 62561, Saudi Arabia; amobark@kku.edu.sa

**Keywords:** clonal hematopoiesis, therapy-related clonal hematopoiesis, therapy-selected clonal hematopoiesis, CHIP, TP53, PPM1D, therapy-related myeloid neoplasms, cancer survivorship, PRISMA-ScR

## Abstract

Background: Clonal hematopoiesis (CH) is characterized by the proliferation of blood cell clones that possess acquired genetic modifications. While therapy-related CH refers to CH detected in the setting of prior or current treatment in cancer populations, therapy-selected CH demands demonstration that treatment preferentially expanded or maintained an extant clone; therapy-induced CH requires evidence of de novo treatment-associated mutation supported by serial sampling and mutational-signature analyses. Objective: To map evidence distinguishing therapy-related, therapy-selected and therapy-induced CH across cancer treatment, including clonal dynamics, genomic features, clinical outcomes and surveillance gaps. Methods: Following Preferred Reporting Items for Systematic Reviews and Meta-Analyses extension for Scoping Reviews (PRISMA-ScR) guidance, PubMed/MEDLINE, Embase, Scopus, Web of Science Core Collection, Cochrane Library and Google Scholar were searched from inception through 1 June 2026, and updated on 15 September 2026. Primary evidence mapping was limited to original human studies. Reviews and conceptual sources were used only for context or reference tracking and were ineligible for the primary evidence map. Narrative synthesis was employed as critical variation existed in sequencing platforms, variant allele frequency thresholds, timepoints of sampling, filtering strategies, and outcome definitions. Results: Of 684 records identified, 213 duplicates were removed and 471 records were screened. Across both pathways, 121 full texts were assessed, 87 excluded and 34 primary reports mapped; contextual citations were outside the included set. Contextual references were used for definitions and interpretation and were excluded from the included-report count. Six domains were deductively established and inductively refined during charting: terminology; therapy-associated mechanisms of clonal selection/expansion; genomic architecture; longitudinal clonal dynamics; clinical consequences; and study design and methodological heterogeneity. Treatment-associated selection or expansion of pre-existing TP53 and PPM1D clones was the most consistently reported mechanism of therapy-related CH, while CHEK2 was inconsistently detected. Pediatric cohorts and mutational-signature analyses also supported de novo mutagenesis with therapy in select contexts. Progression to therapy-related myeloid neoplasm was the most consistent outcome across studies; however, cytopenia, CAR-T-therapy-associated toxicity, cardiovascular events and mortality were inconsistently reported. Conclusion: Current evidence supports a spectrum of mechanisms ranging from selection of pre-existing clones to therapy-induced de novo mutation. Existing literature cannot support universal CH screening recommendations or guide treatment decisions. Prospective serial analyses harmonized on definition, employing sensitive baseline assays with paired germline controls and tumor-contamination checks, are needed to justify clinical implementation and assessment of fixed outcomes.

## Simple Summary

Cancer treatments can affect healthy blood-forming cells as well as cancer cells. Some blood-forming cells acquire genetic changes and produce an expanding group of related cells, a process called clonal hematopoiesis. This review examined 34 original human studies to understand how these cell groups change before, during and after cancer treatment and how they relate to patient outcomes. The strongest evidence showed that treatment can favor the growth of cells carrying genetic changes that were already present before therapy. In some settings, treatment may also cause new genetic changes. Clonal hematopoiesis was associated with an increased risk of subsequent blood cancers, while links with other complications and survival were less consistent. Finding these genetic changes after treatment does not, by itself, prove that treatment caused them. More studies with repeated blood testing are needed to determine who might benefit from monitoring. Current evidence does not support routine testing for every patient or changing effective cancer treatment solely because clonal hematopoiesis is detected.

## 1. Introduction

### 1.1. Background

Increased survival from cancer has led to improved recognition and understanding of late effects of cancer treatment. One serious late effect that clinicians and researchers are learning more about is therapy-related myeloid neoplasms (t-MN). t-MN frequently have poor prognostic cytogenetic and molecular abnormalities.

Clonal hematopoiesis (CH) refers to the expansion of blood cells that are derived from a hematopoietic stem or progenitor cell that acquired a somatic alteration. Clonal hematopoiesis of indeterminate potential (CHIP) refers to leukemia-associated somatic mutations (typically present at a variant allele frequency (VAF) ≥2%) that occur in individuals without hematologic malignancy or another cause for cytopenia. Age-related clonal hematopoiesis frequently stems from mutations in DNA methyltransferase 3 alpha (DNMT3A), TET methylcytosine dioxygenase 2 (TET2), and ASXL transcriptional regulator 1 (ASXL1), which also correlate with increased risk of hematologic malignancy, mortality, and cardiovascular disease [1,2,3,4,5].

Cancer therapy imposes additional selective and mutagenic pressures. Cytotoxic chemotherapy, radiotherapy, conditioning, radionuclide therapy and other treatments can differentially/selectively expand pre-existing clones. The most robust study-level data for this are with tumor protein p53 (TP53) and protein phosphatase, Mg2+/Mn2+ dependent 1D (PPM1D), while data for checkpoint kinase 2 (CHEK2) is more mixed [6,7,8,9,10]. ataxia telangiectasia mutated (ATM) is part of the broader DNA-damage response (DDR) pathway but was not separately characterized in the charted studies.

Three operational definitions are applied. Therapy-related CH is strictly descriptive: CH found in an individual who received cancer therapy. Therapy-selected CH necessitates longitudinal or cross-sample evidence that treatment enriched the relative fitness of a pre-existing clone. Therapy-induced CH necessitates stronger evidence for a de novo lesion, ideally documented absence on an adequately sensitive baseline assay with acquisition of a treatment-consistent mutational signature. A clone found only after treatment is therefore called newly detected by this schema, unless treatment was administered before a sensitive enough baseline could be obtained. Selection pressures appear well supported in many adult cohorts [7,8,10,11]. De novo treatment-related mutagenesis may contribute more substantially to pediatric cohorts and after specific exposures, such as platinum agents [12,13].

The most consistently studied clinical endpoint is progression to t-MN. Other endpoints studied include persistent cytopenia, poor hematopoietic recovery, treatment interruption, cell therapy toxicity, cardiovascular disease (CVD), and overall survival [14,15,16,17,18,19,20,21].

### 1.2. Rationale

The evidence is scattered across treatment settings, sequencing platforms, VAF thresholds, sampling schedules, filtering of germline and tumor-only reads, and definitions of outcomes of interest. These distinctions make it difficult to directly compare the results or discern whether clones that were detected after treatment were therapy-selected, newly detected below a prior limit of detection, or induced by therapy.

The features of CH in cancer and t-MN have been described in existing narrative and conceptual reviews [18,22,23,24,25,26]. Compared with these reviews, this scoping review provides a reproducible evidence map that differentiates between the original human studies and background sources, maps study-level characteristics, and highlights where quantitative synthesis can or cannot be performed. Quantitative synthesis through pooling was not appropriate because the studies included did not measure a common effect across sufficiently similar populations, exposures, assays, VAF thresholds, sampling times, and outcomes.

The review applied a therapy–clone–outcome framework for charting. Extracted from each study were (1) treatment exposure; (2) clone/pathway; (3) longitudinal state (pre-existing selection, newly detected/indeterminate, de novo induction, stability, contraction, progression); and (4) clinical outcome. This schema allowed separation of therapy-specific evidence from broader CH evidence and pinpoint variables for potential meta-analysis.

### 1.3. Aim and Objectives

The purpose of this scoping review was to map clonal hematopoiesis before, during and after cancer treatment, including antecedent clones, clones detected in stem-cell products, pre-infusion CH in cellular-therapy cohorts, treatment-associated clonal evolution, and CH as a modifier of treatment outcomes. Progression from antecedent CH to therapy-related myeloid neoplasms was evaluated as a clinical outcome. Emphasis was placed on clonal selection and evolution, high-risk genotypes, clinical impact, and gaps in detection and surveillance.

Objectives included: (1) definition and reporting of therapy-related clonal hematopoiesis; (2) treatment exposures related to clone emergence or clonal expansion; (3) reporting of high-risk genes/genomic patterns; (4) clonal trajectories longitudinally; (5) clinical outcomes associated with therapy-related or therapy-selected clonal hematopoiesis; and (6) gaps in detection, reporting, screening, and surveillance.

Population–concept–context: The population included patients with cancer or survivors of cancer receiving cancer-directed therapy. The concept was detection of CH before, during or after cancer-directed therapy including clone expansion, detection of a new clone, mutational pattern, VAF change, or progression to myeloid neoplasm. Context was specific to the oncology, hematology, transplantation, cellular therapy, survivorship and cardio-oncology clinical settings. The population–concept–context (PCC) framework is summarized above, and the eligibility criteria are detailed in Section 2.2.

## 2. Materials and Methods

### 2.1. Protocol and Registration

This review was conducted as a scoping review and evidence map and checked against PRISMA-ScR guidance. The protocol was not prospectively registered, which remains a transparency limitation. A written protocol has been deposited in Mendeley Data. Before full-text charting, the author finalized the PCC framework, eligibility criteria, search concepts, charting variables and initial synthesis domains. Codes and subthemes were subsequently refined during charting. During revision, primary-study eligibility was separated from contextual citation use, and four reviewer-suggested eligible reports were integrated into the formal map. The deposition is retrospective and does not establish prospective prespecification. The reporting checklist is provided in Supplementary File S1, Table S4 (PRISMA-ScR checklist).

### 2.2. Eligibility Criteria

#### 2.2.1. Population

Studies reporting data from adults or children with cancer, cancer survivors, or transplant/cellular-therapy recipients where CH was measured prior to, during, or after treatment were included. Pediatric evidence was kept to map age-dependent mechanisms but charted and interpreted separately given that baseline age-related CH is rare and treatment mutational signatures may be stronger. The population–concept–context (PCC) framework and eligibility criteria are summarized in Supplementary File S1, Table S2.

#### 2.2.2. Concept

CH was assessed before, during or after cancer therapy. Therapy-related CH was a descriptive label for CH in a therapy-exposed patient. Therapy-selected CH required detection of the same somatic variant or lineage before exposure and evidence of preferential expansion or persistence after exposure, based on serial clone measurements using comparable assays and a treatment-linked change in relative fitness. Persistence alone or cross-sectional enrichment did not establish selection; exposure-specific enrichment without serial confirmation was interpreted as suggestive. VAF changes were interpreted in relation to assay precision, sampling source and changes in blood-cell composition rather than applying an unsupported universal increase threshold. Therapy-induced CH required a newly acquired lesion after exposure, an adequately sensitive negative pretreatment sample with a documented detection limit, and molecular evidence linking acquisition to treatment, such as a treatment-associated mutational signature. Negative baseline testing cannot exclude an arbitrarily small antecedent clone. Where baseline sampling, sensitivity or molecular attribution was inadequate, post-treatment detection was coded as newly detected/indeterminate rather than induced. CHIP and clonal cytopenia of undetermined significance (CCUS) definitions and VAF thresholds were recorded as reported by each study; VAF ≥ 2% was not imposed universally.

#### 2.2.3. Context

Potentially eligible treatment categories included chemotherapy, radiotherapy, combined chemoradiotherapy, platinum, alkylating agents, topoisomerase inhibitors, poly(ADP-ribose) polymerase (PARP) inhibitors, radionuclide therapy, venetoclax-containing therapy, lenalidomide-containing therapy or myeloma-directed therapy, autologous hematopoietic cell transplant (autologous HCT) or allogeneic hematopoietic cell transplant (allogeneic HCT), chimeric antigen receptor T-cell (CAR-T) therapy, immune checkpoint inhibitors, or any other cancer-directed treatment.

#### 2.2.4. Types of Evidence Sources

**Figure 1:**
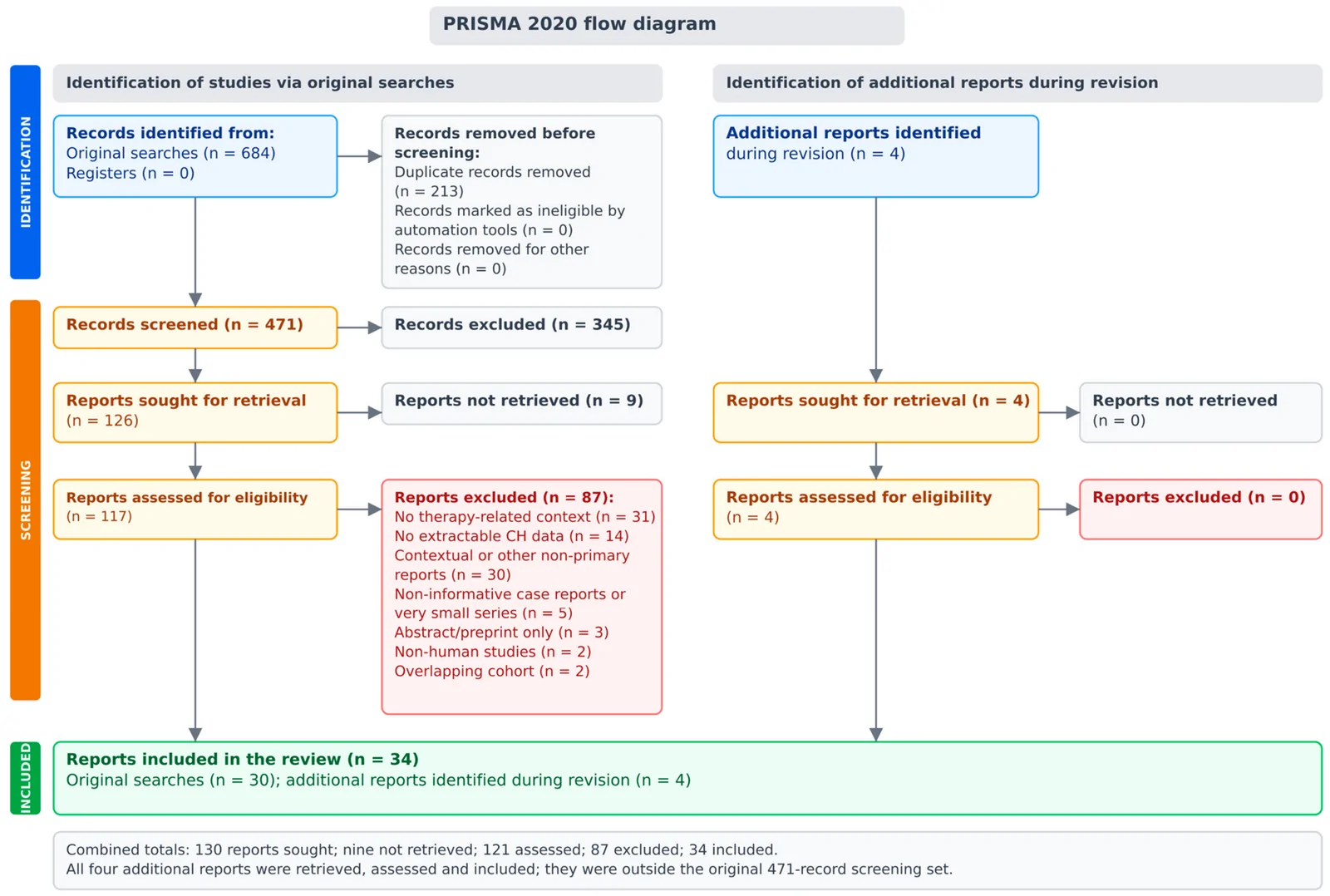
PRISMA flow diagram of report identification and selection. Original searches identified 684 records and contributed 30 included primary reports. Four additional reports identified during revision were all retrieved, assessed and included. Across both pathways, 130 reports were sought for retrieval, nine were not retrieved, 121 were assessed for eligibility, 87 were excluded, and 34 were included. The four revision-stage additions were outside the original 471-record screening set. Contextual citations were not counted as included primary reports.

Only original human studies meeting the population, concept and context criteria were eligible for the formal primary evidence map. Eligible designs included cohort, case–control, serial-sampling, retrospective genomic, transplant and cellular-therapy studies. Reviews, definition papers, general-population studies, commentaries and experimental studies were used solely as contextual citations or for reference tracking; they were not exceptions to primary-study eligibility and were not counted as included studies in Figure 1 or in Table 1A,B. The 11 contextual sources retained during the original selection process are classified separately from the 34 primary reports; eight additional background citations likewise remain outside the primary evidence map. Source classification and evidence-flow mapping are provided in Supplementary File S1, Table S3.

#### 2.2.5. Inclusion Criteria

Studies were eligible if they reported peer-reviewed, English-language human data pertaining to cancer therapy or t-MN that allowed extraction of somatic blood, marrow, or stem-cell-product results regarding genes, variant allele frequency (VAF), clonal dynamics, treatment exposure, or outcome. The English language requirement was applied for practical reasons and may have introduced language/publication bias.

#### 2.2.6. Exclusion Criteria

Studies were excluded if they only investigated age-related CH in healthy individuals without association to cancer therapy, did not provide data on somatic mutations or CH, reported only germline predisposition mutations without analyzing somatic clones, were based only on animals or in vitro experiments, were unpublished conference abstracts or preprints lacking full text, were reviews, editorials or other non-primary sources or reported duplicate data without providing a unique analysis.

#### 2.2.7. Publication Years and Language

No lower publication-date limit was applied. The original database searches were last run on 1 June 2026 which was updated and finalized on 15 September 2026 and incorporated into the final evidence map during revision.

### 2.3. Information Sources

The last original search was conducted on 1 June 2026 for each of the following: PubMed/MEDLINE, Embase, Scopus, Web of Science Core Collection, Cochrane Library and Google Scholar. Reference lists of primary studies and relevant reviews were also checked. For Google Scholar, the first 200 entries returned by the search were examined for potentially eligible reports, using the same primary-study eligibility criteria. Potentially relevant reports were checked in full text and managed with the other retrieved records in EndNote (Clarivate, Philadelphia, PA, USA). Entries beyond the first 200 were not examined. Google Scholar was treated as a supplementary discovery source rather than a comprehensive database search.

### 2.4. Search Strategy

The search strategy was created using terms describing CH, cancer-therapy exposure, clonal evolution/mutational-signature acquisition, and clinical outcomes. Search terms included: clonal hematopoiesis, clonal haematopoiesis, CHIP, clonal hematopoiesis of indeterminate potential, therapy-related clonal hematopoiesis, treatment-related clonal hematopoiesis, therapy-selected clonal hematopoiesis, clonal evolution, clone expansion, variant allele frequency, VAF, TP53, PPM1D, CHEK2, ATM, DNMT3A, TET2, ASXL1, therapy-related myeloid neoplasm, therapy-related AML, therapy-related MDS, chemotherapy, radiotherapy, cancer therapy, cytotoxic therapy, PARP inhibitor, CAR-T, hematopoietic stem-cell transplantation, and autologous transplant.

A representative PubMed search string was: (“clonal hematopoiesis” OR “clonal haematopoiesis” OR CHIP OR “clonal hematopoiesis of indeterminate potential” OR “therapy-related clonal hematopoiesis” OR “therapy-selected clonal hematopoiesis”) AND (chemotherapy OR radiotherapy OR radiation OR “cancer therapy” OR “cytotoxic therapy” OR “PARP inhibitor” OR “CAR-T” OR “hematopoietic stem cell transplantation” OR “autologous transplant”) AND (“clonal evolution” OR “clone expansion” OR “variant allele frequency” OR VAF OR mutation OR “therapy-related myeloid neoplasm” OR “therapy-related AML” OR “therapy-related MDS”).

The search terms and representative PubMed search strategy are reported above; database-specific search strategies are provided in Supplementary File S1, Table S1.

### 2.5. Study Selection

EndNote was used for reference storage, deduplication and management of screening records. Initial screening and charting were performed by the author; a second reviewer, RS (Ravinder Singh Saini), independently verified the work. This verification should be distinguished from duplicate independent screening and extraction performed throughout the review. The original searches identified 684 records; 213 duplicates were removed and 471 records were screened. After exclusion of 345 records at title/abstract screening, 126 reports were sought, nine were not retrieved, and 117 were assessed for primary-study eligibility. Eighty-seven reports were excluded (Supplementary File S2): no therapy-related context (*n* = 31), no extractable CH data (*n* = 14), contextual or other non-primary report (*n* = 30), non-informative case report or very small series (*n* = 5), abstract/preprint only (*n* = 3), non-human study (*n* = 2), or overlapping cohort without a distinct analysis (*n* = 2). The non-primary category includes the 11 contextual sources previously counted separately alongside 19 other background reports. The original-search pathway contributed 30 included primary reports. Four additional eligible reports were identified during revision, and all were retrieved, assessed and included. Across the original searches and revision-stage supplementation, 130 reports were sought for retrieval (126 original-search reports and four additional reports). Nine original-search reports were not retrieved, leaving 121 reports assessed for eligibility (117 original-search reports and four additional reports). Eighty-seven reports were excluded, and 34 primary reports were included (30 from the original searches and four added during revision). The four additional reports were not part of the original 471-record screening set. Contextual citation use did not convert an excluded non-primary report into an included primary study.

Reports from overlapping cohorts were evaluated based on recruitment setting, enrollment period, population enrolled, treatment and sample source. Reports that did not have their own analysis were excluded; when multiple related reports were available, only one was counted unless it provided a unique outcome, time-point, subgroup or molecular analysis. Data from related reports were combined for interpretation. Report numbers were not considered to represent numbers of independent patient populations.

### 2.6. Data Charting and Data Extraction

Study author, year, design, population, treatment information, sample size, age category, sample source, sequencing platform, targeted gene panel, assay limit of detection (LoD), reported variant allele frequency (VAF) threshold, CH definition, sampling time relative to treatment or study enrollment, filtering method used to control tumor contamination, mutations, clonal trajectory analysis methods and results, and reported clinical outcomes were recorded using a standardized charting form (File 3: Study data extraction form, supplied as non-published material for editorial and peer review only). Table 1A shows summary information for studies reporting cytotoxic therapy, radiotherapy, t-MN, transplant, PARP-inhibitor, peptide receptor radionuclide therapy (PRRT), or breast-cancer outcomes. Table 1B shows summary information for studies reporting CAR-T therapy, venetoclax treatment, pediatric outcomes, or performing mechanistic or evolutionary analyses. EndNote was used for bibliographic and record management, while extracted study information was organized in the standardized charting form and manuscript evidence tables. The EndNote version was not recorded. Authors were not contacted for missing information. Unavailable methodological details were recorded as not reported; these entries do not establish that the corresponding procedures were absent.

Clinical outcomes were charted as t-MN, cytopenia/hematopoietic recovery, transplant outcome, CAR-T toxicity, cardiovascular disease, survival, treatment interruption, or another survivorship endpoint. Clonal trajectories were coded as pre-existing selection/expansion, newly detected with induction uncertain, de novo induction supported, stability, contraction/disappearance, progression to t-MN, or indeterminate. The Results are organized into study selection, study characteristics, methodological characteristics, treatment exposures, clonal architecture, longitudinal clonal dynamics, clinical outcomes and evidence gaps; biological explanations and clinical interpretation are presented in the Discussion.

### 2.7. Data Synthesis

Qualitative synthesis was conducted. Meta-analysis and VAF-threshold sensitivity analyses were not possible for most studies as they failed to report effect estimates or stratified data across commonly used VAF cutoffs. Additionally, pooling across studies would be clinically meaningless given important differences between studies in population, exposure, platform, assay sensitivity, baseline sampling, germline/tumor filtering, and outcome definition. A meta-analysis may be possible in the future when published studies report common endpoints and threshold-stratified estimates; meta-regression can explore sources of heterogeneity including VAF cutoff, platform, sample source, age group, therapy class, and follow-up duration. Study-specific prevalence estimates were not directly compared across assays unless they were performed in the same sampling context.

Six broad synthesis domains were specified deductively from the review objectives and charting framework before full-text charting: terminology; therapy-associated selection/expansion mechanisms; genomic architecture; longitudinal clonal dynamics; clinical consequences; and study design and methodological heterogeneity. They were not prospectively registered before screening. During charting, codes and subthemes were refined inductively within these domains; this refinement was distinct from changing primary-study eligibility. These domains served as evidence-mapping categories rather than formal qualitative constructs. No dedicated qualitative-analysis software was used.

### 2.8. Methodological Limitations and Temporal Inference

Formal design-specific risk-of-bias appraisal was considered but was not undertaken because the purpose of this scoping review was to map the characteristics and interpretive limits of heterogeneous evidence rather than estimate pooled effects or establish clinical recommendations. No study was excluded on methodological-quality grounds. Instead, methodological limitations were mapped descriptively using the study-level information already charted in Table 1A,B. Supplementary File S1, Table S5, consolidates sampling and temporality, molecular measurement, and variant-attribution information, and explains the resulting constraints on interpretation. This mapping is not a validated appraisal instrument, an overall quality rating, or an assessment of certainty of evidence. No numerical score was calculated.

Temporal inference was rated as strong if identical (or at least equally sensitive) molecular testing was available both before and after treatment. If serial sampling was incomplete or if a valid comparator group was reported, evidence was rated as moderate. When sampling was limited to cross-sectional or post-treatment data only, evidence was rated limited. Studies were marked as not assessable if there was insufficient reporting of timing or sensitivity of testing. Categories above refer to clonal timing only. Pretreatment sampling from a CAR-T cohort does not exclude other causes but can substantiate an association with toxicity that follows CAR-T therapy without proving CAR-T selected or induced CH. Assay thresholds were defined separately from analytical detection limits. Where methodological details were unspecified, we maintained these as not reported rather than making assumptions. Confounding by age, prior and cumulative treatment exposure, baseline CH and marrow reserve, as well as outcome definitions, follow-up and competing risks were discussed where applicable to the charted findings; we did not re-extract or formally rate these criteria for each study.

## 3. Results

### 3.1. Study Selection

The original searches identified 684 records. After removal of 213 duplicates, 471 records were screened and 345 were excluded. Of 126 reports sought for retrieval, nine were not retrieved; 117 were assessed for eligibility and 87 were excluded, leaving 30 included primary reports. Four additional reports identified during revision were retrieved, assessed and included. Across both pathways, 130 reports were sought, 121 were assessed, 87 were excluded and 34 primary reports were included (Figure 1). Excluded reports and reasons are listed in Supplementary File S2. Contextual citations remain outside the included primary evidence map. The four additional reports were identified through targeted supplementation rather than a comprehensive rerun of all databases.

### 3.2. Study Characteristics

The final evidence map included 34 original human reports published in 2015–2026: 23 cohorts (67.6%), three case–control reports (8.8%), three clinical case series (8.8%) and five clinical/mechanistic or genomic reports (14.7%). Waldschmidt was categorized as a retrospective cohort, selected-case serial analysis. Two reports were pediatric-specific (5.9%) and 32 adult/mixed or not pediatric-specific (94.1%). Eight reports were published in 2015–20(20), 14 reports in 20(21)–20(23) and 12 reports in 20(24)–20(26). Table 1A,B lists all 34 reports. Table 1C summarizes the profile of these reports. Counts are based on primary reports, not confirmed independent cohorts. Treatment context groups are based on the principal setting of each report; outcome domains are not mutually exclusive. Studies proposed by reviewers [31,43,44,45] are included in the final denominator of 34 reports. Contextual citations were not included. Each addition was screened for unique data/analyses; the presence of two similar reports does not necessarily indicate unique patient populations.

Temporal-inference categories: strong = adequate pretreatment and post-treatment molecular samples using comparable, sufficiently sensitive assays; moderate = partial serial sampling or a credible comparison group; limited = cross-sectional or post-treatment-only sampling; not assessable = timing or assay sensitivity insufficiently reported. These categories describe temporal inference only; variant filtering and overall study quality are reported separately. Studies are ordered by publication year and alphabetically by first author within each year.

VAF caution: Prevalence and clone-size estimates should not be compared directly across studies using different VAF thresholds, assay sensitivities, sample sources or sampling times.

Temporal-inference categories are defined in the footnote to Table 1A. Studies are ordered by publication year and alphabetically by first author within each year. Abbreviations are defined in the manuscript abbreviation list.

Primary treatment/context groups are mutually exclusive descriptive assignments. Percentages may not sum to 100 because of rounding.

### 3.3. Methodological Characteristics

Conventional targeted panels/exome methods were described in 16 reports (47.1%), error-corrected or duplex targeted sequencing in 5 (14.7%), whole-genome sequencing in 2 (5.9%), and integrated, multimethod or poorly described approaches in 11 (32.4%). Arends and Al-Sawaf contributed error-corrected targeted analyses, Rappa contributed conventional targeted sequencing data, and Waldschmidt contributed integrated WGS/targeted data analyzed at the case-level. Methods varied by sample source, error-correction and variant attribution procedures (Table 1A,B).

Minimum variant allele frequencies (VAFs) used as inclusion thresholds were at least 2% in 5 reports, at least 1% in 4 reports, at least 0.5% in 2 reports (one of which used an exploratory threshold of 0.5%), and assay-specific cutoffs in 6. We were unable to identify a stated threshold for inclusion in 17 reports (50.0%). Analytical calling thresholds were differentiated from thresholds used for inclusion in the CH (Analyses performed on the bone marrow sample at baseline by Arends used a 0.1% calling floor but included variants in the CH at a 1% threshold). Temporal inferences were strong in 11 reports (32.4%), moderate in 13 (38.2%), and limited in the remaining 10 (29.4%) reports. Partial baseline/sample-set sampling resulted in moderate ratings for Al-Sawaf, Rappa and Waldschmidt. Features discussed in Table 1D and Supplementary File S1, Table S5, were described but not used to inform an overall quality grade.

Definitions of CH/CHIP were not consistent across reports. Definitions of clinical endpoints varied similarly. We note that reporting of treatment dose, treatment schedule, cumulative dose exposure and variant attribution also varied. Not reported refers to an item that was left unspecified in the charting process rather than a procedure that was shown to be absent. Clinical outcome methods and adjustment for potential confounders were beyond the scope of our re-extraction and were not rated for all reports.

VAF categories reproduce the thresholds charted in Table 1A,B. Variant filtering is recorded separately at study level and was not inferred when unreported.

### 3.4. Treatment Exposures

Primary contexts included general cytotoxic, radiotherapy or t-MN settings in eight reports (23.5%); transplant/myeloma in four (11.8%); PARP-inhibitor-containing therapy in four (11.8%); breast-cancer systemic therapy in three (8.8%); pediatric multimodal therapy in two (5.9%); CAR-T in nine (26.5%); venetoclax/CLL treatment in two (5.9%); and PRRT and lenalidomide in one report each (2.9%). Al-Sawaf spanned multiple trial regimens, and for simplicity of mutually exclusive counting, Al-Sawaf was placed into venetoclax/CLL. Note that contexts are not mutually exclusive study settings versus single exposures (Table 1C).

Exposures were defined as platinum agents, alkylating agents, topoisomerase inhibitors, radiotherapy or chemoradiation, transplant conditioning and maintenance treatment in general [6,7,8,9,10,11,12,13,14,15,16,17,19,20,21,27,28,29,30,32,35,37]. The nine reports mapping CAR-T predominantly examined CH present prior to infusion in previously treated patients [34,36,38,39,40,41,42]. No eligible therapy-specific original study of immune checkpoint inhibitors was discovered in the systematic map. Exposure-specific mechanisms can be interpreted from the Discussion and Table 2.

### 3.5. Clonal Architecture

TP53 and PPM1D were the most commonly reported genes across studies showing enrichment/expansion after genotoxic therapy [6,9,10,17,30,32]. CHEK2 observations were mixed, and ATM was not independently characterized across the charted studies. Other CH genes reported include DNMT3A, TET2 and ASXL1. B-cell lymphoma 2 (BCL2)-associated X protein (BAX)-mutated myeloid cells were described in the longitudinal venetoclax trial [35].

TP53 mutant antecedent clones and evolving t-MN were observed in multiple studies, including reports of complex karyotypes or chromosome 5/7 anomalies among the relevant t-MN cases [11,37]. Across the charted studies, ASXL1 mutations were not uniquely related to chromosome 5/7 abnormalities. Germline predisposition and tumor-derived mutations were analyzed separately from somatic hematopoietic clones if the methodologies enabled (Table 1A,B).

Eight studies included explicit overall CH/CHIP frequencies across the charted cohorts. The observed ranges per study were 2.7% to 58.2%; previously we reported 62% vs. 27% in a case–control setting. Added to the previously summarized estimates are Arends (51% CH; 35% DDR-CH), Al-Sawaf (58.2% at VAF ≥ 0.5%; 35.5% CHIP at ≥2 mutations)) and Rappa (39/104, 37.5% at ≥1 mutation). Waldschmidt’s 12/179 (6.7%) was post-CAR-T myeloid clonality frequency and is not equivalent to systematic CH prevalence. Estimates are not pooled or ranked in order (Table 1E).

### 3.6. Longitudinal Clonal Dynamics

Trajectories comprised expansion, persistence, stability, contraction/disappearance, acquisition of additional mutations, and progression to t-MN [7,8,10,11,14,15,16,17,30,32]. Adult longitudinal data demonstrated expansion of existing TP53 and PPM1D mutant clones following genotoxic therapy [9,10,17,30,32]. Pediatric paired-sample and mutation signature data provided supporting evidence of treatment-related mutagenesis in selected cases [12,13]. Adult and pediatric data were mapped independently. Arends et al. demonstrated preferential expansion of DDR clones; Alsawaf et al. demonstrated BAX/U2 small nuclear RNA splicing factor 1 (U2AF1) mutant clone expansion with blunted proliferative capacity after venetoclax therapy; Waldschmidt et al. described relationships between prior marrow clones and late post-CAR-T myeloid neoplasms; and Rappa et al. characterized TP53/ASXL1 mutant clone expansion in contrast to DNMT3A/PPM1D contraction over time [31,43,44,45].

Serial chemotherapy and radiation reports described phenotypic evolution as an increase in TP53 mutant clone prevalence or volume [17]. The PRRT manuscript described patients with existing or newly identified DDR gene mutations at follow-up [28], while the venetoclax case series described BAX mutant myelopoiesis and other myeloid dysplasia [35]. Pre-existing blood or stem cell product mutations were associated with later development of myeloid malignancies in subsets of patients [7,8,11,14,15,16].

Where pretreatment sampling or baseline sensitivity was inadequate, first post-treatment detection was retained as newly detected/indeterminate. Cross-sectional enrichment and post-treatment detection alone were not classified as demonstrated induction. Study-level temporal categories are reported in Table 1A,B,D; the four reviewer-suggested included reports [31,43,44,45] are charted in the tables and discussed in Section 4.1.

### 3.7. Clinical Outcomes

Fifteen studies evaluated t-MN or secondary myeloid malignancy, 12 evaluated cytopenia/hematologic recovery, nine evaluated CAR-T toxicity/recovery, nine evaluated survival/mortality, seven evaluated cancer response/disease control, one evaluated cardiovascular events and two evaluated treatment interruption (Table 1E). Totals reflect domains evaluated, even if results were negative, not positive outcomes detected. Al-Sawaf’ Richter transformation was a lymphoid outcome and was not included in the t-MN total.

Antecedent CH and subsequent t-MN have been linked in the context of cytotoxic treatment and transplantations [7,8,10,11,14,15,16,37]. Persistent cytopenia or delayed hematologic recovery was described after PRRT, breast-cancer chemotherapy, CAR-T treatment, venetoclax and transplantation [14,27,28,30,35,41,42]. In these cases, associations were described, but causation by CH was not established.

CAR-T results were mixed. Some studies detected no obvious relationship between pre-existing CH and cytokine release syndrome (CRS), immune effector cell-associated neurotoxicity syndrome (ICANS), response or survival [34,38,39,42]; others found associations with severe neurotoxicity, grade ≥ 2 CRS, delayed recovery, or hematologic toxicity [36,40,41]. One treatment-specific human cohort found increased late risk of heart failure and ischemic cardiovascular disease among patients with solid tumors and CHIP [33]. Non-treatment-specific general-population and experimental cardiovascular results were not considered treatment-specific human outcomes. Addition of the Rappa cohort left outcomes neutral for several domains when CH was grouped into a single category. However, adverse hematologic/inflammatory outcomes were linked to TP53-mutant CH [44]. Waldschmidt reported selected serial cases from within a larger179-patient cohort and described heavy prior melphalan use [45].

Survival and cancer-control findings varied across treatment contexts. No pooled effect estimates were calculated. Clinical interpretation of the mapped associations is presented in the Discussion and Table 3.

Outcome domains are non-mutually exclusive. Reported prevalence estimates are not directly comparable across assays, thresholds, sample sources, populations and sampling times. Among cohort and case–control reports with an explicit total sample size in the charting, sample sizes ranged from 40 to 24,439.

### 3.8. Evidence Gaps

The final map includes 2 pediatric-specific reports, 1 PRRT report, 2 venetoclax/CLL-context reports, 1 therapy-specific cardiovascular cohort, and 2 reports addressing treatment interruption. No therapy-specific immune-checkpoint-inhibitor studies were eligible for inclusion. Most of the 9 CAR-T reports used pre-existing CH as an outcome modifier; serial analyses of selected subsets provided insight into clone dynamics but did not isolate a causal CAR-T effect.

Baseline assay sensitivity, variant allele frequency (VAF) thresholds, variant filtering, cumulative treatment exposure, and clinical outcome definitions were unevenly reported. Consequently, the evidence map did not resolve whether some newly detected clones represented expansion below a prior detection limit versus new mutation acquisition. Similarly, comprehensive study-level evaluation of confounder adjustment and clinical outcome ascertainment was not available from the existing charting methods.

Prospective evidence evaluating benefit from universal CH screening or treatment alterations based solely on CH was not identified in the mapped reports. Each of the four reviewer-suggested reports was added to the formal map, but their targeted identification does not constitute nor replace a comprehensive updated search. Discussion of these gaps in research priorities and potential biological explanations is provided in the Discussion.

## 4. Discussion

### 4.1. Principal Conclusions and Their Evidential Basis

The 34-report primary map comprised 23 cohorts, three case–control studies, three case series and five clinical–mechanistic or genomic investigations. It supports two recurring observations: treatment-associated expansion of antecedent DDR-mutant clones in serial adult studies, and an association between antecedent CH and later myeloid malignancy in several treatment settings. These conclusions concern observed trajectories and associations; they do not establish that any detected clone was caused by treatment or that CH testing improves outcomes. Counts below refer to reports, with possible cohort overlap, and are not measures of formal evidence certainty.

Five adult cohort reports with serial or prospective molecular sampling provide direct observations relevant to selection after genotoxic treatment: Bolton, Nead, Arends, Mayerhofer and Morganti [10,17,30,31,32]. Two pediatric genomic reports address a different question—treatment-associated mutagenesis [12,13]. Fifteen mapped reports addressed t-MN or secondary myeloid malignancy, whereas 12 addressed cytopenia/recovery, nine CAR-T toxicity/recovery, nine survival/mortality and one cardiovascular events. The larger and more consistent t-MN literature must therefore be distinguished from the smaller or discordant evidence for other outcomes (Table 1A–E).

**Figure 2:**
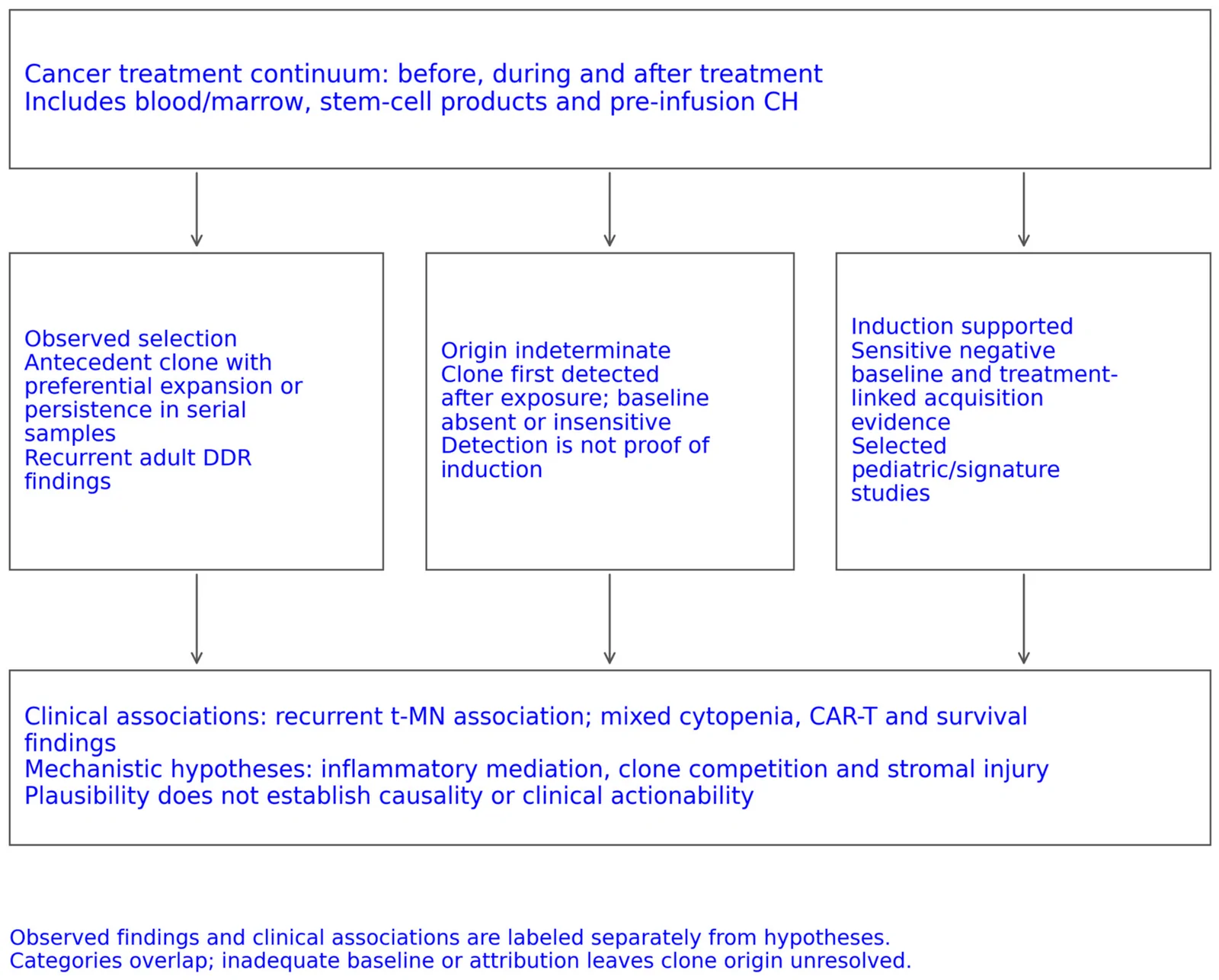
Clonal hematopoiesis across the cancer treatment continuum. The diagram distinguishes observed selection, newly detected clones of indeterminate origin, and induction supported in selected settings; these are interpretive states rather than mutually exclusive biological compartments or overall quality grades. Selection requires antecedent clone evidence and preferential expansion/persistence. Induction requires a sufficiently sensitive baseline and treatment-linked acquisition evidence. Solid boxes summarize observed findings or their evidential requirements; the lower panel explicitly separates clinical associations from mechanistic hypotheses. TP53/PPM1D selection is recurrent in adult genotoxic-treatment reports, whereas pediatric genomic reports support mutagenesis in selected settings. The nine CAR-T reports include pre-infusion outcome-modifier cohorts and two selected serial analyses [44,45], without establishing general CAR-T-induced CH. CH, clonal hematopoiesis; DDR, DNA-damage response; t-MN, therapy-related myeloid neoplasm; CAR-T, chimeric antigen receptor T-cell therapy.

Here, robust denotes a repeatedly observed pattern across relevant designs, not a formal Grading of Recommendations Assessment, Development and Evaluation (GRADE) judgment. The most defensible conclusions are that pre-existing clones can expand during treatment and that antecedent CH is associated with later t-MN in selected populations. General de novo induction, an independent CAR-T effect, a PARP-inhibitor-specific causal effect, and actionable prediction of toxicity, cardiovascular events or mortality remain incompletely established or hypothesis-generating. Figure 2 summarizes the distinction between clonal selection, newly detected clones of uncertain origin, and therapy-induced mutation across the cancer treatment continuum.

#### 4.1.1. Adult Selection and Pediatric Mutagenesis

The five adult cohort reports [10,17,30,31,32] provide temporally ordered observations, complemented by one paired antecedent/t-MN genomic report [11] and two clinical–experimental mechanistic reports concerning PPM1D and lenalidomide/TP53 [9,37]. These designs address different parts of the selection argument: serial sampling documents change, paired samples link antecedent clones to later disease, and functional experiments test relative fitness. Their convergence supports selection in specific exposures, but sequential therapies and differences in blood-cell composition can still complicate attribution of an observed VAF increase. Findings from the two prospective breast-cancer cohorts [29,32] also show that therapy-exposed CH is not uniformly associated with adverse clinical outcomes.

The two pediatric reports comprise a genomic proof-of-concept study in neuroblastoma and a mutational-signature cohort in childhood-cancer survivors [12,13]. Their paired or serial genomic analyses support treatment-associated mutation acquisition in selected contexts. The rarity of age-related CH in children changes the competing explanation compared with adults, but neither rarity nor a negative baseline test alone establishes induction. Two reports cannot define the frequency of induction across pediatric cancers or justify extrapolation to adult treatment settings. Table 2 distinguishes the exposure-specific mechanistic evidence; its descriptive support labels are not risk-of-bias or certainty grades.

Interpretive key: Supported = replicated longitudinal, comparative or functional evidence; Suggestive = limited or partially confounded evidence; Uncertain/insufficient = current studies cannot reliably attribute the mechanism.

Important: Selection evidence indicates preferential expansion or persistence of a pre-existing clone. Induction requires evidence of a de novo lesion, including an adequately sensitive negative baseline sample and molecular evidence linking acquisition to treatment, such as a treatment-associated mutational signature. Newly detected post-treatment CH should not automatically be classified as therapy-induced.

The originally mapped CAR-T studies mainly assessed pre-existing CH and clinical outcomes. Additional longitudinal reports describe TP53-mutant clonal expansion after infusion, without establishing de novo CAR-T-induced CH [44,45]. Abbreviations are defined in the manuscript abbreviation list.

#### 4.1.2. Contradictory Findings in CAR-T and PARP-Inhibitor Settings

The nine mapped CAR-T reports were all cohorts; five had limited, three moderate, and one strong temporal inference (Table 1B). All except Hudyma’s tested CH present before infusion, so established clinical associations concern outcome modification rather than demonstration of CAR-T-induced CH. Panagiota’s serial, error-corrected multicenter cohort found no consistent toxicity or survival association [39], while severe ICANS was reported with the axicabtagene cohort of Saini [36], higher grade ≥ 2 CRS with Goldsmith’ mixed CD19/B-cell maturation antigen (BCMA) cohort [40], and hematologic toxicity or delayed recovery with BCMA CARs by Avigan and Gustine [41,42]. These studies tested different endpoints and treatment populations, rather than being interchangeable replications.

Plausible explanations for discordance between studies include assay and cohort differences. Panagiota had serial blood sampling and used a VAF threshold of 1%, while Goldsmith and Gustine used thresholds of 2% and Gustine also analyzed pre-infusion marrow. CAR target and product, lymphoma versus myeloma patients, prior treatment and baseline marrow reserve, clone genotype, and definitions of toxicity differed. Smaller cohorts may simply lack power to detect genotype-specific effects. Testing multiple genes and outcomes can lead to unstable associations. These potential explanations for disagreement were not tested by meta-regression but should be considered hypotheses rather than demonstrated causes. An overall null association with CH does not exclude gene-specific associations, nor does the latter prove a universal CH-based rule for toxicity.

The four mapped PARP-inhibitor publications consisted of two trial-associated serial/clini-cal cohorts [19,31] and two selected case series [20,21]. Pre-platinum treatment, relapsed disease, total amount of therapy and duration of PARP-inhibitor exposure are necessarily linked to clone detection and risk of t-MNs. The cohorts described either concurrent or temporally sequenced associations, while the case series lack denominators to estimate excess incidence. Enrichment of pre-existing TP53 mutations does not distinguish platinum selection from a subsequent PARP-inhibitor exposure; prolonged exposure could also preferentially detect patients that survive or respond long enough to qualify for continued treatment. Available study designs can therefore not distinguish an independent drug-specific cause. Attribution limitations to a single agent were discussed in a related commentary [46].

Two later added reports on CAR-T included post-infusion genotype-specific kinetics, describing TP53 clonal expansion in this setting as well [44,45]. However, meaningful exposure to prior melphalan, stem cell transplantation, maintenance therapy or lymphodepletion in these settings limits clarity on attribution to CAR-T specifically. The EUDARIO study, with 423 serial samples from 103 patients [31], describes DDR-clone kinetics during treatment with a combination of carboplatin, a PARP inhibitor, and a heat shock protein 90 (HSP90)-inhibitor; combination exposure similarly confounds simple attribution to a single agent. Finally, the trial-associated CLL publication added with longitudinal samples from 620 patients supports a temporal relationship and potential function for selection of BAX-associated mutations during venetoclax-containing regimens [43]. All four reports were carried over to the final list of 34 mapped publications, while reviewer-suggested origins are preserved. Table 3 differentiates clinical associations from actionable science.

Interpretive key: Strong = repeated and broadly concordant therapy-specific evidence; Moderate = evidence from several studies with important heterogeneity or confounding; Limited = few therapy-specific studies or indirect evidence; Mixed = materially inconsistent results across comparable clinical settings.

Important: Association does not establish causality or clinical actionability. VAF, gene identity, sample source, assay sensitivity, timing, blood counts, treatment exposure and competing clinical factors should be interpreted together.

Cardiovascular evidence: General-population studies of CH and atherosclerosis provide mechanistic context only and are not counted as primary therapy-specific outcome evidence in this table.

Abbreviations: BCMA, B-cell maturation antigen; CAR-T, chimeric antigen receptor T-cell therapy; CCUS, clonal cytopenia of undetermined significance; CH, clonal hematopoiesis; CHIP, clonal hematopoiesis of indeterminate potential; CRS, cytokine-release syndrome; DDR, DNA-damage response; ICANS, immune effector cell-associated neurotoxicity syndrome; PARP, poly(ADP-ribose) polymerase; PRRT, peptide receptor radionuclide therapy; t-MN, therapy-related myeloid neoplasm; VAF, variant allele frequency; cfDNA, cell-free DNA; PBMC, peripheral blood mononuclear cells; MRD, measurable residual disease; LoD, limit of detection; U2AF1, U2 small nuclear RNA auxiliary factor 1; CD19/CD138, cluster of differentiation 19/138.

#### 4.1.3. Survivorship, Competing Risks and Biological Plausibility

Survivorship and sampling can affect both clone prevalence and outcome associations. Cohorts sampled after treatment, at transplantation or before CAR-T include patients who survived and remained eligible for those procedures; they may underrepresent early deaths, severe toxicity or patients without available specimens. Repeat molecular sampling may preferentially include patients with prolonged follow-up or clinical abnormalities. These mechanisms can distort comparisons with pretreatment populations and limit generalizability. They are potential design biases, not biases formally demonstrated or quantified for every mapped report.

For t-MN and cardiovascular outcomes, death from the primary cancer is a competing event; relapse and subsequent treatment can alter both exposure and outcome risk. Analyses that treat competing deaths as ordinary censoring may overestimate cumulative incidence, while conventional survival associations may reflect cancer severity, age, treatment burden or comorbidity. Nine reports addressed survival/mortality and 15 myeloid malignancy, but competing-risk methods and time-varying exposures were not systematically appraised here. The review therefore cannot determine whether CH independently changes absolute long-term risk across treatment settings. In future comparative studies, time zero, the timing of CH ascertainment and subsequent treatment should be explicit to limit survivorship and immortal-time bias.

Biological plausibility and demonstrated association require separate evidence. The two clinical–experimental mechanistic reports [9,37] support relative fitness under specific drugs; the two pediatric genomic reports [12,13] support treatment-associated mutational processes. By contrast, experimental cardiomyopathy findings [47] and other preclinical studies [48] are contextual sources, not additional human outcome cohorts. General-population cardiovascular studies and reviews [49,50] provide additional context, not treatment-specific outcome evidence. Only one therapy-specific biobank cohort addressed cardiovascular events [33]. An inflammatory mechanism can therefore help explain a candidate association without demonstrating mediation, an exposure-specific causal effect or benefit from intervention.

Germline predisposition, tumor-derived variants and changes in sample composition provide additional alternative explanations for apparent CH signals. TP53, CHEK2 and ATM findings require somatic-versus-germline attribution; BRCA1 DNA repair associated (BRCA1) and BRCA2 DNA repair associated (BRCA2) chiefly represent inherited cancer-predisposition context in the PARP-inhibitor reports [19,20]. The charted differences in filtering and VAF thresholds prevent treating all positive variants or equivalent VAF values as biologically comparable. General-population risk models [51,52,53] provide relevant context, but their predictive performance cannot be assumed in heavily treated cancer survivors.

#### 4.1.4. Conclusions That Remain Hypothesis-Generating

The t-MN association comprises the largest evidence body with 15 mapped publications following cohort, case–control, paired genomic, and selected case-series designs. Serial continuity supports the interpretation of an antecedent clone, although case selection and overlapping populations preclude considering every publication as independent validation. Support for cytopenia/recovery (12 reports) and CAR-T consequences (nine cohorts) is more disparate; the cardiovascular inference is based on one therapy-specific cohort and mortality risk inference on nine reports spanning different clinical contexts. Overall, there is no mapped evidence supporting the use of a general screening approach, one-size-fits-all actionable VAF threshold, or treatment modification based on CH alone. Mechanistic hypotheses including inflammation, competition with the emerging clone, or stromal injury remain conjecture until experimentally evaluated in humans.

### 4.2. Comparison with Existing Literature

Table 4 provides a structured comparison by research question, populations, exposures, search coverage, primary-study count and synthesis method. Jahn’s earlier review [25] emphasizes therapy-selected CH and t-MN mechanisms; Rondeau [54] examines cancer-treatment pressures and clonal evolution; Vanner [55] uses a broader clinical CH/CCUS management framework; and Croitoru [56] pools clinical outcomes in solid tumors. These reviews answer related but distinct questions, so agreement on DDR-clone selection represents conceptual convergence rather than independent validation using entirely separate evidence.

Notes: Not reported denotes an unspecified review-level search cutoff or eligible primary-study count; reference-list length was not used as a proxy. *For Rondeau et al., scope was verified from the publisher summary and PubMed record; unavailable full-text search/count details are explicitly identified. Publication date is not a search cutoff. Comparator reviews are contextual citations, not additions to the primary evidence map. CH, clonal hematopoiesis; CCUS, clonal cytopenia of undetermined significance; HR, hazard ratio; OR, odds ratio; Quality in Prognostic Factor Studies (QUIPS), Quality in Prognostic Factor Studies; t-MN, therapy-related myeloid neoplasm.

Croitoru’s 21-study solid-tumor meta-analysis used QUIPS appraisal and pooled hazard ratios (HRs)/odds ratios (ORs) [56], whereas the present review maps 34 human reports across adult, pediatric, transplant and cellular-therapy settings without formal bias appraisal or pooling. Its contribution is the separation of exposure, clone, temporal state and outcome, rather than a larger count or a new claim that therapy selects CH. Search periods also differ: the original searches end on 1 June 2026, with four targeted reviewer-suggested September additions integrated into the final map. Comparator search/count details unavailable for Rondeau remain marked as unreported. Study-level overlap was not quantified, and differences in reported totals cannot establish greater completeness or novelty.

### 4.3. Clinical and Scientific Implications

The evidence supports research stratification by gene, clonal trajectory, exposure history and outcome, but not routine treatment selection based on CH. Fifteen reports addressing myeloid malignancy identify a recurrent risk association; they do not demonstrate that screening or changing effective cancer treatment improves survival. Nine CAR-T cohorts, four PARP-inhibitor reports and one cardiovascular cohort provide insufficiently consistent or causal evidence for treatment-specific management rules. This review therefore proposes no universal screening schedule, surveillance interval or treatment-withholding threshold.

Clinical actionability requires more than mechanistic coherence or statistical association: a model must show discrimination, calibration and incremental value in the relevant treatment population, and an intervention must demonstrate benefit relative to its harms. None of those requirements is established by the present descriptive map. Incidental CH and unexplained cytopenia remain clinical questions governed by patient-specific assessment and applicable practice guidance; this review should not be read as a clinical guideline.

### 4.4. Strengths of the Review

The review separates 34 eligible primary human reports from contextual citations and documents the provenance of four reviewer-suggested additions. Its study-level tables preserve sampling, assay, exposure and outcome information, and the Results distinguish observed findings from interpretation. Independent verification by RS provides an additional check on the author’s initial work. The descriptive methodological-limitations map makes unspecified information visible without converting temporal categories into overall quality grades. These features improve transparency but do not remove the review limitations.

### 4.5. Limitations

Limitations include English-only inclusion, initial screening and charting by one author with subsequent independent verification by RS, rather than duplicate independent review throughout, lack of prospective protocol registration, and absence of a formal risk-of-bias assessment. Study designs, platforms, VAF thresholds, sample sources, germline/tumor filtering, timing, and outcome definitions were heterogeneous; formal VAF sensitivity analysis and meta-analysis were therefore not possible. Selection, newly detected clones, and de novo induction could not always be distinguished. These constraints limit causal and clinical inference. Four reviewer-suggested reports were incorporated into the final map through targeted identification, rather than a complete database update; eligible reports may therefore remain undiscovered. The descriptive methodological-limitations map does not replace a formal risk-of-bias assessment and cannot establish overall study quality or certainty of clinical associations. It is based on the existing charted information; confounder adjustment, outcome ascertainment, follow-up completeness and competing-risk methods were not systematically evaluated across all studies. Consequently, the temporal-inference categories must not be interpreted as evidence-quality grades. Google Scholar was limited to the first 200 entries, and its query/session settings were not fully documented. Missing information was not pursued through author contact. The September supplementation does not establish a fully updated systematic evidence map.

### 4.6. Future Research Directions

The next priority is prospective treatment-specific cohorts with pretreatment and serial molecular sampling, comparable error-corrected assays and documented limits of detection. Adult and pediatric populations require separate analyses. Matched-tumor and nonhematopoietic controls should be incorporated where feasible to clarify variant origin. Such designs can test the selection and induction hypotheses more directly than post-treatment prevalence comparisons.

Outcome studies should define time zero and sample ascertainment, record cumulative and time-varying exposures, distinguish CAR-T products and disease contexts, and model death and relapse as competing events where appropriate. Adequately powered genotype-specific analyses are needed to investigate contradictory CAR-T findings; comparisons accounting for prior platinum and combined regimens are needed for PARP-inhibitor attribution. Standardized outcomes and assay thresholds would permit focused synthesis within comparable strata rather than pooling heterogeneous treatment contexts.

Clinical implementation remains a separate research question. Prospective validation must establish whether CH adds useful prediction beyond routine clinical variables, and intervention studies must test whether monitoring or management changes improve patient outcomes. Feasibility, incidental germline findings, patient burden and access to follow-up should be evaluated alongside benefit, without assuming that detecting more clones improves care.

## 5. Conclusions

Across 34 mapped human reports, serial adult observations most consistently support treatment-associated expansion of antecedent DDR-mutant clones, while two pediatric genomic reports support treatment-associated mutagenesis in selected settings. Fifteen reports addressed subsequent myeloid malignancy, the most recurrent adverse clinical association. These findings do not imply that every clone detected after therapy was induced by it.

CAR-T toxicity, cytopenia/recovery, cardiovascular and survival associations remain heterogeneous or limited. The four reviewer-suggested reports extend the final map without establishing a comprehensive database search update. The evidence does not establish benefit from universal CH screening, a single actionable VAF threshold or altering effective cancer therapy solely because CH is detected. Prospective exposure-specific studies and clinical-utility evaluations are required.

## Figures and Tables

**Table 1:** (**A**) Characteristics of original studies in cytotoxic therapy, radiotherapy, t-MN, transplant, PARP-inhibitor, PRRT, and breast-cancer settings. (**B**) Characteristics of original studies in CAR-T, venetoclax, pediatric, and mechanistic or evolutionary settings. (**C**) Study design, primary therapy context, publication period and age focus (N = 34). (**D**) Assay and temporal-sampling characteristics (N = 34). (**E**) Outcome domains and reported prevalence (N = 34).

(**A**)								
**Study**	**Design and Population**	**Cancer Therapy**	**Sample Timing**	**Sequencing Method**	**VAF Threshold**	**Temporal Inference**	**Variant Filtering**	**Main Finding**
Wong et al., 2015 [11]	Paired antecedent/t-MN genomic study	Cytotoxic therapy before t-AML	Antecedent and t-AML samples	WES with targeted validation	Assay-specific threshold	Strong	Paired-sample somatic validation	TP53 mutations were present before therapy in some patients and expanded during t-AML evolution
Coombs et al., 2017 [6]	Institutional cohort; >8800 patients	Prior cytotoxic therapy and/or radiotherapy	Single blood sample in relation to tumor profiling	MSK-IMPACT blood–tumor paired analysis	Not reported	Limited	Matched tumor comparison performed; residual tumor/germline misclassification remained possible	CH occurred in approximately 25%; TP53 and PPM1D were enriched after cytotoxic exposure
Gillis et al., 2017 [8]	Nested case–control; 13 t-MN cases and 56 controls	Chemotherapy	Sequential samples	Targeted sequencing and WES	Not reported	Moderate	Not reported; germline classification was uncertain for some genes	CHIP was detected in 62% of cases and 27% of controls; some antecedent clones expanded at t-MN
Takahashi et al., 2017 [7]	Retrospective case–control; t-MN cases and matched controls	Chemotherapy and/or radiotherapy	Pretreatment or pre-t-MN samples	Targeted sequencing	Not reported	Moderate	Not reported	Antecedent CH was more common in patients who subsequently developed t-MN
Bolton et al., 2020 [10]	Large cohort with sequential sampling; 24,439 patients	radiotherapy, platinum agents and topoisomerase II inhibitors	Baseline and follow-up samples in a subset	MSK-IMPACT CH calling and t-MN modeling	Assay-specific threshold	Strong	Matched tumor comparison and study-defined bioinformatic filtering	Radiotherapy, platinum and topoisomerase II inhibitors were associated with selection of TP53, PPM1D and CHEK2 clones
Mouhieddine et al., 2020 [27]	Transplant cohort in multiple myeloma	Induction, autologous HCT, maintenance and prior therapy	Stem-cell product or blood before/around autologous HCT	Targeted sequencing	Not reported	Moderate	Not reported	CH was associated with adverse post-transplant outcomes in multiple myeloma
Soerensen et al., 2020 [14]	Nested case–control after autologous HCT; 36 cases and 36 controls	autologous HCT and prior cytotoxic therapy	Leukapheresis product before autologous HCT with clinical follow-up	NGS plus immunophenotyping	Not reported	Moderate	Not reported	CH in the collected product and aberrant CD7 expression were associated with later t-MN
Kwan et al., 2021 [19]	Clinical-trial-linked ovarian cancer cohort	Rucaparib after prior platinum exposure	Baseline/pre-rucaparib blood	Targeted blood sequencing	Not reported	Moderate	TP53 tumor/germline distinction assessed; detailed filtering method not reported	Pre-existing TP53-variant CH was associated with secondary myeloid-neoplasm risk
Navitski et al., 2021 [20]	Case series; BRCA-associated ovarian cancer	Platinum chemotherapy before planned PARP-inhibitor maintenance	Before PARP-inhibitor decision; blood and/or marrow	NGS	Not reported	Limited	Germline context considered; somatic-filtering method not reported	Myeloid-driver alterations were identified before PARP-inhibitor decisions
Oliveira et al., 2022 [21]	Case series of PARP-inhibitor-treated patients with MDS/AML	Olaparib, rucaparib, niraparib, veliparib or talazoparib	At MDS/AML evaluation	Marrow morphology, cytogenetics and NGS	Not reported	Limited	Somatic versus germline classification was uncertain	Complex karyotypes and TP53 alterations were common
Singh et al., 2022 [28]	Serial case series; neuroendocrine tumors	Peptide receptor radionuclide therapy	Baseline and serial follow-up samples	100-gene targeted panel	VAF ≥ 1%	Moderate	Not reported	Baseline CH was common; TP53, CHEK2 and PPM1D clones expanded in serial cases
Gibson et al., 2023 [29]	Prospective cohort; 878 young women with breast cancer	Chemotherapy, radiotherapy and/or endocrine therapy	Longitudinal blood samples	Targeted sequencing	VAF ≥ 2%	Strong	Population-frequency/bioinformatic filtering; detailed criteria not reported	CHIP prevalence was 2.7%; most variants involved DNMT3A or TET2, without a strong adverse signal
Mayerhofer et al., 2023 [30]	Prospective serial cohort; 40 older women with breast cancer	Neoadjuvant or adjuvant chemotherapy	Baseline, 6 weeks and 12 weeks	Targeted sequencing	Not reported	Strong	Population-frequency/bioinformatic filtering; detailed criteria not reported	TP53 and PPM1D clones emerged or expanded; CH was associated with neutropenia and dose holds
Arends et al., 2024 [31]	Trial-linked serial cohort; 103 relapsed ovarian-cancer patients; 423 samples	Carboplatin with PARP/HSP90 inhibitors; previous PARP/platinum exposure	Baseline and serial on-treatment whole blood/plasma	Error-corrected 72-gene panel; selected single-cell analyses	CH VAF ≥ 1%; calling floor 0.1%; serial retention ≥ 0.8%	Strong	VAF/population-frequency germline filtering; paired blood/cfDNA attribution; selected single-cell validation	CH in 51%; DDR-CH in 35%; preferential TP53/PPM1D expansion; antecedent clones linked to t-MN in two investigated cases
Awada et al., 2024 [15]	Retrospective cohort; 1507 autologous-HCT patients plus comparators	Autologous HCT, radiotherapy and multiple chemotherapy lines	Pre-HCT and follow-up/diagnostic samples	Serial marrow molecular profiling	Not reported	Strong	Not reported	Pre-existing CH predicted post-HCT t-MN; CH-derived t-MN had worse outcomes
Morganti et al., 2024 [32]	Prospective cohort; 380 breast-cancer patients, 614 samples	Chemotherapy or endocrine therapy	Baseline and longitudinal follow-up	Error-corrected targeted sequencing	VAF ≥ 2%; exploratory threshold ≥ 0.5%	Strong	Error-corrected bioinformatic filtering; detailed germline/tumor criteria not reported	Chemotherapy was not associated with new CHIP at 2%, but low-level TP53 CH was detected after chemotherapy
Nead et al., 2024 [17]	Prospective serial cohort; lung or esophageal cancer	Chemoradiation	Before and after chemoradiation	Error-corrected duplex sequencing	Assay-specific threshold	Strong	Duplex error correction; detailed germline/tumor filtering not reported	TP53 mutation number and clone size increased after therapy and were associated with shorter OS
Yan et al., 2024 [16]	Autologous-transplant cohort in Hodgkin lymphoma	Prior therapy and autologous HCT	Pre-autologous HCT material with longitudinal follow-up	Targeted sequencing	Not reported	Moderate	Not reported	Pre-existing CH was associated with subsequent t-MN risk
Shyr et al., 2026 [33]	Biobank cohort; 8004 patients with solid tumors	Chemotherapy, radiotherapy and/or immunotherapy	Biobank sample with longitudinal cardiovascular follow-up	Whole-genome sequencing; 58-gene CHIP definition	VAF ≥ 2%	Limited	Population-frequency/bioinformatic germline filtering; detailed criteria not reported	CHIP was associated with higher 10-year risks of heart failure and ischemic cardiovascular disease
(**B**)								
**Study**	**Design and Population**	**Cancer Therapy**	**Sample Timing**	**Sequencing Method**	**VAF Threshold**	**Temporal Inference**	**Variant Filtering**	**Main Finding**
Hsu et al., 2018 [9]	Clinical and experimental mechanistic study	Cisplatin and other DNA-damaging agents	Clinical samples with functional experiments	Clinical mutation analysis plus cell and mouse assays	Not reported	Limited	Not reported	PPM1D-truncating mutations promoted resistance to DNA damage and clonal fitness after therapy
Coorens et al., 2021 [12]	Pediatric genomic proof-of-concept study	Neuroblastoma therapy including platinum, myeloablation and radiotherapy	Serial samples	WGS plus deep targeted sequencing	Assay-specific threshold	Strong	Matched-sample analysis; detailed filtering criteria not reported	Pediatric findings supported therapy-associated mutagenesis in addition to selection
Miller et al., 2021 [34]	CAR-T cohort	CD19-directed CAR-T after prior therapy	Pre-infusion CH assessment with post-infusion outcomes	Targeted blood sequencing	Not reported	Limited	Not reported	Pre-existing CH was common; clinical associations were exploratory
Bertrums et al., 2022 [13]	Pediatric mutational-signature cohort	Childhood cancer therapy	Paired/serial samples in relation to therapy and t-MN	WGS with mutational-age/signature analysis	Assay-specific threshold	Strong	Matched-sample and mutational-signature analysis	Therapy-associated mutagenesis contributed to pediatric t-MN
Blombery et al., 2022 [35]	CLL cohort with longitudinal myeloid analysis	Venetoclax	Baseline and serial treatment samples	Targeted sequencing	Not reported	Moderate	Not reported	BAX-mutated myelopoiesis and other myeloid abnormalities emerged during venetoclax therapy
Saini et al., 2022 [36]	Large B-cell lymphoma CAR-T cohort	Axicabtagene ciloleucel after prior therapy	Pre-infusion CH assessment with post-infusion outcomes	Targeted blood sequencing	Not reported	Limited	Tumor-origin review reported; detailed filtering criteria not reported	Pre-existing CH was associated with severe ICANS in this cohort
Sperling et al., 2022 [37]	Clinical and experimental mechanistic study	Lenalidomide after prior therapy	Clinical specimens plus functional experiments	Genomic and functional analyses	Not reported	Moderate	Not reported	Lenalidomide favored TP53-mutant hematopoiesis and t-MN development
Teipel et al., 2022 [38]	Aggressive-lymphoma CAR-T cohort	CD19-directed CAR-T after prior therapy	Samples collected around CAR-T treatment	Targeted sequencing	Study-defined CHIP threshold	Moderate	Not reported	CH prevalence varied over treatment; clinical associations were limited
Panagiota et al., 2023 [39]	Multicenter cohort; 110 B-NHL or ALL patients	Anti-CD19 CAR-T after substantial prior therapy	Pre-infusion and serial post-infusion samples	Error-corrected targeted sequencing	VAF ≥ 1%	Strong	Error-corrected sequencing; detailed germline/tumor filtering not reported	Pre-existing CH was common; no consistent association with CRS, ICANS, cytopenia, response, PFS or OS was observed
Goldsmith et al., 2024 [40]	Cohort; 62 NHL or multiple-myeloma patients	CD19- or BCMA-directed CAR-T after prior therapies	Pre-infusion assessment	108-gene targeted exome sequencing (~1000×)	VAF ≥ 2%	Limited	Not reported	Pre-existing CH occurred in 24%; grade ≥ 2 CRS was more frequent among patients with CH
Avigan et al., 2025 [41]	BCMA CAR-T cohort with inflammatory and toxicity endpoints	BCMA-directed CAR-T after substantial prior therapy	Pre-infusion CH assessment with post-infusion outcomes	CH testing plus inflammatory markers	Not reported	Limited	Not reported	CH and inflammation were associated with hematologic toxicity and secondary myeloid malignancies
Gustine et al., 2025 [42]	Retrospective cohort; 104 patients with multiple myeloma	BCMA CAR-T (ide-cel or cilta-cel)	Pre-infusion marrow with outcome follow-up	Targeted exome sequencing of marrow	VAF ≥ 2%	Limited	Tumor-derived TP53 variants excluded when origin was uncertain	CHIP occurred in 55%; no clear response or survival signal was identified, but hematopoietic recovery was delayed
Al-Sawaf et al., 2026 [43]	Trial-linked CLL cohort; 620 patients from CLL12/CLL14; selected functional analyses	Ibrutinib/placebo; venetoclax-obinutuzumab or chlorambucil-obinutuzumab	CLL12 baseline; CLL14 partial baseline and serial treatment/follow-up samples	Error-corrected targeted sequencing; selected WES/single-cell analyses	VAF ≥ 0.5%; CHIP also summarized at ≥2%	Moderate	CD19-negative sorted cells in CLL12; MRD-informed attribution in CLL14; selected single-cell validation	CH in 58.2%; BAX/U2AF1 selection under venetoclax, PPM1D under chlorambucil; neutropenia and treatment-dependent survival/PFS associations
Rappa et al., 2026 [44]	Observational CAR-T cohort; 104 NHL/myeloma patients; longitudinal samples in 26/39 CH-positive patients	CD19- or BCMA-directed CAR-T after prior therapies and lymphodepletion	Around lymphodepletion; serial post-infusion PBMCs in a subset	Conventional 74-gene targeted panel; serum proteomics	Variants VAF ≥ 1%; proportions also reported at ≥2%	Moderate	Flow cytometry excluded circulating malignancy; tumor/marrow comparison showed no variant overlap; germline strategy incompletely detailed	CH-associated variants in 39/104 (37.5%); TP53/ASXL1 expansion; overall CH neutral for several outcomes, with TP53-specific adverse/inflammatory associations
Waldschmidt et al., 2026 [45]	Multicenter retrospective CAR-T cohort; 179 myeloma patients; 12 myeloid-clonality cases selected for detailed study	Mainly BCMA CAR-T; extensive prior melphalan, autologous HCT and lenalidomide	Pretreatment material available in 8/12 cases; serial CD138-negative marrow in 10/12	WGS in selected cases/controls plus informed targeted sequencing	Driver mutation VAF ≥ 2% for CCUS; separate analytical LoD not reported	Moderate	CD138-negative marrow; WGS/melphalan-signature analysis; comprehensive germline filtering not detailed in the letter	12/179 with myeloid clonality (5 AML, 3 MDS, 4 CCUS); antecedent TP53 expansion and melphalan signatures; prior exposure confounds attribution
(**C**)								
**Characteristic**			**Category**				**Studies, *n* (%)**	
Primary therapy/context			General cytotoxic, radiotherapy or t-MN				8 (23.5)	
			Transplant or myeloma				4 (11.8)	
			PARP inhibitor				4 (11.8)	
			Breast-cancer systemic therapy				3 (8.8)	
			Pediatric multimodal therapy				2 (5.9)	
			CAR-T				9 (26.5)	
			PRRT				1 (2.9)	
			Venetoclax				2 (5.9)	
			Lenalidomide				1 (2.9)	
Study design			Cohort				23 (67.6)	
			Case–control				3 (8.8)	
			Clinical case series				3 (8.8)	
			Clinical–mechanistic or genomic				5 (14.7)	
Publication period			2015–2020				8 (23.5)	
			2021–2023				14 (41.2)	
			2024–2026				12 (35.3)	
Age focus			Pediatric-specific				2 (5.9)	
			Adult, mixed or not pediatric-specific				32 (94.1)	
(**D**)								
**Characteristic**			**Category**				**Studies, *n* (%)**	
Sequencing approach			Conventional targeted panel/exome				16 (47.1)	
			Error-corrected or duplex targeted				5 (14.7)	
			WGS				2 (5.9)	
			Integrated, multimethod or incompletely specified				11 (32.4)	
Reported VAF threshold			VAF ≥ 2%				5 (14.7)	
			VAF ≥ 1%				4 (11.8)	
			VAF ≥ 2% with exploratory threshold ≥ 0.5%				1 (2.9)	
			VAF ≥ 0.5%				1 (2.9)	
			Assay-specific threshold				6 (17.6)	
			Not reported				17 (50.0)	
Temporal-inference category			Strong				11 (32.4)	
			Moderate				13 (38.2)	
			Limited				10 (29.4)	
(**E**)								
**Outcome Domain**			**Studies Reporting Domain, *n***				**Descriptive Finding**	
t-MN or secondary myeloid malignancy			15				Most consistently adverse domain	
Cytopenia, hematologic toxicity or recovery			12				Heterogeneous across therapies	
CAR-T toxicity or recovery			9				Mixed associations	
Overall survival or mortality			9				Mixed; confounding common	
Cancer response or disease control			7				No consistent association	
Cardiovascular events			1				Limited therapy-specific human evidence	
Treatment interruption			2				Two cohorts reporting treatment interruption, with differing findings	
Reported CH/CHIP prevalence			8 reports with explicit overall CH/CHIP proportions				Overall-cohort CH/CHIP estimates 2.7–58.2%; case–control 62% vs. 27%. Waldschmidt’s 6.7% myeloid-clonality frequency is a different endpoint.	

Abbreviations: AML, acute myeloid leukemia; CH, clonal hematopoiesis; CHEK2, checkpoint kinase 2; CHIP, clonal hematopoiesis of indeterminate potential; DNMT3A, dna methyltransferase 3 alpha; HCT, hematopoietic cell transplantation; MDS, myelodysplastic neoplasm/myelodysplastic syndrome; NGS, next-generation sequencing; OS, overall survival; PPM1D, protein phosphatase, mg2+/mn2+ dependent 1d; TET2, tet methylcytosine dioxygenase 2; TP53, tumor protein p53; VAF, variant allele frequency; WES, whole-exome sequencing; t-AML, therapy-related acute myeloid leukemia; t-MN, therapy-related myeloid neoplasm; cfDNA, cell-free DNA; PBMC, peripheral blood mononuclear cells; MRD, measurable residual disease; LoD, limit of detection; U2AF1, U2 small nuclear RNA auxiliary factor 1; CD19/CD138, cluster of differentiation 19/138. ALL, acute lymphoblastic leukemia; B-NHL, B-cell non-Hodgkin lymphoma; BCMA, B-cell maturation antigen; CAR-T, chimeric antigen receptor T-cell therapy; CH, clonal hematopoiesis; CHIP, clonal hematopoiesis of indeterminate potential; CLL, chronic lymphocytic leukemia; CRS, cytokine release syndrome; ICANS, immune effector cell-associated neurotoxicity syndrome; NHL, non-hodgkin lymphoma; OS, overall survival; PFS, progression-free survival; TP53, tumor protein p53; VAF, variant allele frequency; WGS, whole-genome sequencing; t-MN, therapy-related myeloid neoplasm; cfDNA, cell-free DNA; PBMC, peripheral blood mononuclear cells; MRD, measurable residual disease; LoD, limit of detection; U2AF1, U2 small nuclear RNA auxiliary factor 1; CD19/CD138, cluster of differentiation 19/138. CAR-T, chimeric antigen receptor T-cell therapy; PARP, poly(ADP-ribose) polymerase; PRRT, peptide receptor radionuclide therapy; t-MN, therapy-related myeloid neoplasm; cfDNA, cell-free DNA; PBMC, peripheral blood mononuclear cells; MRD, measurable residual disease; LoD, limit of detection; U2AF1, U2 small nuclear RNA auxiliary factor 1; CD19/CD138, cluster of differentiation 19/138. VAF, variant allele frequency; WGS, whole-genome sequencing. CAR-T, chimeric antigen receptor T-cell therapy; CH, clonal hematopoiesis; CHIP, clonal hematopoiesis of indeterminate potential; t-MN, therapy-related myeloid neoplasm; cfDNA, cell-free DNA; PBMC, peripheral blood mononuclear cells; MRD, measurable residual disease; LoD, limit of detection; U2AF1, U2 small nuclear RNA auxiliary factor 1; CD19/CD138, cluster of differentiation 19/138.

**Table 2:** Therapy contexts and proposed clonal mechanisms.

Therapy Context	Recurrent Genes/Pathways	Selection Evidence	Induction Evidence	Mechanistic Interpretation	Key Studies
Platinum chemotherapy	TP53, PPM1D, CHEK2 and other DDR genes	Supported: serial adult cohorts show enrichment or expansion of pre-existing DDR-mutant clones after exposure.	Suggestive: pediatric mutational-signature studies support acquisition of treatment-associated mutations in selected settings.	Both mechanisms may occur. Selection appears prominent in adults with pre-existing CH, whereas direct mutagenesis may contribute more in children and after highly mutagenic exposure.	[9,10,12,13,19,30,32]
Alkylating agents and myeloablative conditioning	TP53, PPM1D; adverse cytogenetic patterns in subsequent t-MN	Suggestive to supported: antecedent CH in transplant products or pretreatment samples may persist or expand through conditioning and marrow stress.	Uncertain: direct mutagenesis is biologically plausible, but the mapped clinical studies rarely distinguish it from prior-treatment effects.	Genotoxic injury and a hematopoietic bottleneck may favor resistant pre-existing clones. Prior therapy, mobilization and conditioning are difficult to separate.	[14,15,16,27]
Topoisomerase II inhibitors	DDR genes; therapy-associated rearrangements in t-MN contexts	Suggestive: exposure-associated enrichment of DDR clones has been reported in mixed therapy cohorts.	Suggestive: topoisomerase II inhibitors are mutagenic, but direct induction of CH is not established by most clinical CH cohorts.	Selection and de novo mutagenesis may coexist. Serial baseline sampling and treatment-specific mutational signatures are required for attribution.	[10,13]
Radiotherapy and chemoradiation	TP53 most consistently; PPM1D and CHEK2 in mixed cohorts	Supported: serial studies report post-treatment expansion or increased detection of DDR-mutant clones, particularly TP53.	Uncertain: a post-treatment-only clone cannot be considered induced without a sensitive negative baseline sample and signature evidence.	Radiation may deplete susceptible hematopoietic cells and increase the relative fitness of resistant DDR-mutant clones.	[10,15,17]
PARP inhibitors	TP53; PPM1D and ATM reported in broader DDR contexts	Suggestive: pre-existing TP53-variant CH has been associated with subsequent myeloid neoplasms in PARP-inhibitor-treated ovarian cancer.	Uncertain: direct PARP-inhibitor induction is not separable from prior platinum exposure in current studies.	The apparent association is strongly confounded by prior platinum therapy, treatment duration and cumulative genotoxic exposure.	[19,20,21,31]
Peptide receptor radionuclide therapy	TP53, PPM1D and CHEK2	Suggestive: a small serial study reported expansion of baseline DDR-mutant clones.	Uncertain: evidence is insufficient to establish de novo induction.	Targeted radionuclide exposure may create radiation-like selection pressure, but the evidence base is small and requires replication.	[28]
Autologous hematopoietic cell transplantation	TP53, PPM1D; DNMT3A and TET2 in broader CH	Supported for risk association: pre-existing CH in collected products or pretransplant samples predicts t-MN in several cohorts.	Not established: transplantation itself cannot be isolated from prior chemotherapy, mobilization and conditioning.	Transplantation is a composite clinical setting rather than a single mutagenic exposure. Clonal bottlenecks and reinfusion of antecedent clones may contribute.	[14,15,16,27]
Lenalidomide and myeloma-directed therapy	TP53 most relevant; broader CH may include DNMT3A, TET2 and PPM1D	Supported mechanistically for selection: clinical and experimental data indicate preferential fitness of TP53-mutant hematopoiesis during lenalidomide exposure.	Not established as a general de novo CH-induction mechanism.	Drug-specific selection should be distinguished from the effects of prior cytotoxic therapy, autologous HCT and cumulative marrow injury.	[27,37]
Venetoclax	BAX-mutated myelopoiesis; other myeloid-driver alterations	Suggestive: longitudinal observations indicate treatment-associated selection of BAX-mutant myeloid populations.	Not established.	This is an emerging, drug-specific example of clonal selection. Findings from CLL cohorts should not be generalized to all cancer populations.	[35,43]
CAR-T therapy	Pre-existing CH involving DNMT3A, TET2, ASXL1, PPM1D or TP53; inflammatory pathways	Not established as CAR-T-mediated CH selection. Most studies assessed CH present before infusion.	Not established. CAR-T is not supported as a direct CH-inducing exposure.	Current evidence concerns effect modification: pre-existing CH may influence inflammatory signaling, CRS, ICANS, cytopenia or hematopoietic recovery. Findings are mixed and confounded by prior therapy.	[34,36,38,39,40,41,42,44,45]
Immune checkpoint inhibitors	No reproducible therapy-specific CH gene pattern identified in the mapped evidence	Insufficient evidence.	Insufficient evidence.	Inflammatory interactions are biologically plausible, but original human evidence was too limited for a mechanistic conclusion.	No eligible therapy-specific original study identified
Endocrine therapy comparator	Predominantly age-related CH genes such as DNMT3A and TET2	No consistent evidence of strong therapy-specific selection.	Not established.	Endocrine therapy may function as a less-genotoxic comparator, but age, prior therapy and baseline CH must be considered.	[29,32]

Abbreviations: ASXL1, asxl transcriptional regulator 1; ATM, ataxia telangiectasia mutated; CAR-T, chimeric antigen receptor T-cell therapy; CH, clonal hematopoiesis; CHEK2, checkpoint kinase 2; CLL, chronic lymphocytic leukemia; CRS, cytokine release syndrome; DDR, dna-damage response; DNMT3A, dna methyltransferase 3 alpha; HCT, hematopoietic cell transplantation; ICANS, immune effector cell-associated neurotoxicity syndrome; PARP, poly(ADP-ribose) polymerase; PPM1D, protein phosphatase, mg2+/mn2+ dependent 1d; TET2, tet methylcytosine dioxygenase 2; TP53, tumor protein p53; t-MN, therapy-related myeloid neoplasm; cfDNA, cell-free DNA; PBMC, peripheral blood mononuclear cells; MRD, measurable residual disease; LoD, limit of detection; U2AF1, U2 small nuclear RNA auxiliary factor 1; CD19/CD138, cluster of differentiation 19/138.

**Table 3:** Clinical outcome domains and interpretation.

Outcome Domain	Therapy-Specific Evidence	Relevant CH Features	Consistency of Evidence	Clinical Interpretation	Key Studies
Therapy-related myeloid neoplasms (t-MN)	Antecedent CH and expansion of pre-existing clones before t-MN have been reported after cytotoxic therapy and in autologous-transplant settings. Paired samples support lineage continuity in a subset of patients.	TP53 or PPM1D involvement; multiple mutations; rising VAF; cytopenia; adverse cytogenetic or mosaic chromosomal alterations; abnormal stem-cell product	Strongest and most consistent outcome domain	The recurring association supports prospective risk-prediction research. No universal screening threshold or benefit from treatment alteration is established.	[7,8,10,11,14,15,16,31,44,45]
Overall survival and mortality	Associations with survival have been reported in selected solid-tumor, chemoradiation and transplant cohorts, but estimates are strongly influenced by cancer type, age, treatment burden and competing risks.	TP53 expansion; CH-derived t-MN; multiple/high-risk mutations; impaired marrow reserve	Moderate and heterogeneous	Therapy-specific prognostic models require adjustment for cancer characteristics, exposure history and competing mortality; CH alone is not a validated prognostic rule.	[6,10,15,17,31,43,44]
Cytopenia, myeloid abnormalities and hematopoietic recovery	Persistent cytopenia, myeloid abnormalities or delayed recovery have been reported after breast-cancer chemotherapy, PRRT, venetoclax, CAR-T and transplantation. Causality is not established.	DDR-gene clones; TP53; PPM1D; BAX-mutated myelopoiesis; DNMT3A/TET2; baseline marrow reserve; coexisting cytopenia	Moderate but inconsistent across settings	The association is compatible with reduced hematopoietic reserve but remains multifactorial. Prospective analyses must account for disease, infection, inflammation, cumulative therapy and evolving t-MN.	[14,27,28,30,31,35,41,42,43,44,45]
CAR-T-associated toxicity and recovery	Most studies assessed CH present before infusion. Some reported associations with CRS, ICANS, hematologic toxicity or delayed recovery; others found no clear relationship with toxicity, response or survival.	Pre-existing CH; DNMT3A, TET2, ASXL1, PPM1D or TP53; inflammatory markers; prior therapy burden	Mixed and emerging	These findings concern modification of outcomes by pre-existing CH. Inflammatory mediation and an independent CAR-T effect require direct testing; no CH-based toxicity-management rule is established.	[34,36,38,39,40,41,42,44,45]
Cardiovascular disease	One cancer-therapy-specific human cohort reported higher long-term risks of heart failure and ischemic cardiovascular disease among patients with CHIP. Experimental mouse evidence provides mechanistic context but is not therapy-specific human outcome evidence.	CHIP status; inflammatory CH pathways; TP53-mutant CH; age; baseline cardiovascular risk; cardiotoxic therapy exposure	Limited therapy-specific human evidence	One therapy-specific human cohort supports further cardio-oncology investigation. General-population and mouse studies provide plausibility rather than direct treatment-specific causal evidence.	[33]
Cancer-treatment decision-making	Pre-existing TP53 CH or other high-risk features have been examined around PARP-inhibitor therapy, transplantation and other genotoxic exposures. No prospective trial demonstrates benefit from altering effective cancer treatment solely because CH is detected.	TP53; multi-hit or rising DDR clone; CCUS; unexplained cytopenia; multiple mutations; prior treatment burden	Insufficient for treatment-changing recommendations	No prospective trial demonstrates benefit from modifying effective cancer treatment solely on the basis of CH. The evidence does not establish a treatment-withholding threshold.	[15,19,20,31,43]
Primary cancer response and disease control	Studies in young women with breast cancer and in CAR-T cohorts generally reported neutral or inconsistent associations between CH and cancer response, progression-free survival or overall survival.	Gene identity; clone size and trajectory; inflammatory phenotype; cancer type; therapy context	Limited and inconsistent	CH is not validated for treatment selection or response prediction. Future models require therapy-specific prospective validation.	[29,31,34,39,42,43,44]

Abbreviations: ASXL1, asxl transcriptional regulator 1; CAR-T, chimeric antigen receptor T-cell therapy; CCUS, clonal cytopenia of undetermined significance; CH, clonal hematopoiesis; CHIP, clonal hematopoiesis of indeterminate potential; CRS, cytokine release syndrome; DDR, dna-damage response; DNMT3A, dna methyltransferase 3 alpha; ICANS, immune effector cell-associated neurotoxicity syndrome; PPM1D, protein phosphatase, mg2+/mn2+ dependent 1d; PRRT, peptide receptor radionuclide therapy; TET2, tet methylcytosine dioxygenase 2; TP53, tumor protein p53; VAF, variant allele frequency; t-MN, therapy-related myeloid neoplasm; cfDNA, cell-free DNA; PBMC, peripheral blood mononuclear cells; MRD, measurable residual disease; LoD, limit of detection; U2AF1, U2 small nuclear RNA auxiliary factor 1; CD19/CD138, cluster of differentiation 19/138.

**Table 4:** Comparison with existing reviews of clonal hematopoiesis in cancer and clinical care. Panel (**A**) summarizes scope; panel (**B**) summarizes search coverage, primary-study counts and synthesis frameworks.

(**A**) **Research question, populations and treatment settings.**			
**Review**	**Research Question**	**Included Populations**	**Treatment Settings**
Jahn et al., 2023 [25]	How does therapy-selected CH contribute to t-MN?	Cancer-exposed CH and t-MN; de novo myeloid neoplasms as background.	Antineoplastic exposure, cytotoxic therapy and transplantation; emphasis on myeloid evolution.
Rondeau et al., 2026 [54]	How do cancer therapies shape CH evolution and affect outcomes?	Hematological and nonhematological cancers.	Chemotherapy, radiotherapy, immunotherapy and targeted therapy.
Vanner et al., 2026 [55]	How should CH testing, risk assessment and monitoring inform clinical care?	Human CH/CCUS across oncology and non-oncology clinical settings.	Cancer therapy within a broader framework of CH diagnosis and management.
Croitoru et al., 2026 [56]	Is CH associated with survival, cardiovascular events and mortality?	Adults with solid tumors; studies reporting comparative effect estimates.	Solid-tumor cohorts across treatment and sampling contexts.
Present review	What human evidence distinguishes clonal selection, uncertain emergence and induction, and links these states to outcomes?	Adults and children; solid and hematological cancers; antecedent CH, stem-cell products, pre-infusion CH and subsequent t-MN.	Cytotoxic/radiotherapy, transplant, CAR-T, PARP inhibitors, PRRT, venetoclax and lenalidomide.
(**B**) **Search coverage, primary-study counts and synthesis framework.**			
**Review**	**Search Period/Cutoff**	**Primary Studies**	**Synthesis Framework**
Jahn et al., 2023 [25]	Not reported.	Not reported as an eligible primary-study total.	Narrative synthesis of t-MN genomics, therapy-selected CH, mechanisms and clinical implications.
Rondeau et al., 2026 [54]	Not specified in the accessible abstract/indexed record. * Published July 2026.	Not specified in the accessible abstract/indexed record. *	Mechanism-focused review of selective pressures, clonal evolution and clinical consequences.
Vanner et al., 2026 [55]	Databases from inception to December 2025; additional reference and conference searches.	Not reported as an eligible primary-study total.	Clinical vignettes and a practice framework for diagnosis, risk stratification, surveillance and interventions.
Croitoru et al., 2026 [56]	PubMed, Scopus and Web of Science from inception to February 2026.	21 primary studies.	PRISMA systematic review; QUIPS assessment; pooled HRs/ORs with heterogeneity and sensitivity analyses.
Present review	Six sources through 1 June 2026; four reviewer-suggested reports added on 15 September 2026 and integrated during revision.	34 included primary human reports: 30 original-search reports plus four reviewer-suggested additions; contextual citations excluded.	PRISMA-ScR mapping using treatment → clone/pathway → longitudinal state → outcome; six thematic domains; no pooled estimates.

Abbreviations: CAR-T, chimeric antigen receptor T-cell therapy; CCUS, clonal cytopenia of undetermined significance; CH, clonal hematopoiesis; PARP, poly(ADP-ribose) polymerase; PRRT, peptide receptor radionuclide therapy; t-MN, therapy-related myeloid neoplasm; cfDNA, cell-free DNA; PBMC, peripheral blood mononuclear cells; MRD, measurable residual disease; LoD, limit of detection; U2AF1, U2 small nuclear RNA auxiliary factor 1; CD19/CD138, cluster of differentiation 19/138. CH, clonal hematopoiesis; HRs, hazard ratios; ORs, odds ratios; PRISMA-ScR, Preferred Reporting Items for Systematic Reviews and Meta-Analyses extension for Scoping Reviews; QUIPS, Quality in Prognostic Factor Studies; t-MN, therapy-related myeloid neoplasm. * For Rondeau et al., information was obtained from the publisher’s summary and PubMed record. The search cutoff and number of included primary studies could not be verified from the accessible information and are therefore reported as unspecified. The publication date should not be interpreted as the search cutoff.

## Data Availability

All data charted for this scoping review are presented in the article and its published Supplementary Materials. No new patient-level dataset was generated. The written review protocol has been deposited in Mendeley Data. Supplementary File S1 contains Tables S1–S5: database-specific search strategies, the PCC framework and eligibility criteria, source classification and evidence-flow mapping, the PRISMA-ScR checklist, and descriptive study-level mapping of methodological limitations. Supplementary File S2 lists the 87 reports excluded after full-text assessment and their reasons for exclusion. File S3 contains the study data extraction form, including guidance on clonal trajectories and temporal inference, and is supplied for editorial and peer review only; it is not part of the published Supplementary Materials.

## Supplementary Materials

The following supporting information can be downloaded at: https://www.mdpi.com/article/10.3390/cancers18203398/s1, Supplementary Materials: Supplementary File S1: Supplementary tables—Table S1: Database-specific search strategy; Table S2: PCC framework and eligibility criteria; Table S3: Classification and evidence-flow mapping of all cited references; Table S4: PRISMA-ScR checklist; Table S5: Descriptive study-level mapping of methodological limitations. Supplementary File S2: Excluded studies—reports excluded after full-text assessment (*n* = 87) and reasons for exclusion. File S3: Study data extraction form is not intended for publication and is excluded from the published Supplementary Materials.

## Abbreviations

The following abbreviations are used in this manuscript:

ALL	Acute lymphoblastic leukemia
AML	Acute myeloid leukemia
ASXL1	ASXL transcriptional regulator 1
ATM	Ataxia telangiectasia mutated
BCMA	B-cell maturation antigen
BRCA1	Breast cancer gene 1
BRCA2	Breast cancer gene 2
CAR-T	Chimeric antigen receptor T-cell therapy
CBC	Complete blood count
CCUS	Clonal cytopenia of undetermined significance
CH	Clonal hematopoiesis
CHEK2	Checkpoint kinase 2
CHIP	Clonal hematopoiesis of indeterminate potential
CLL	Chronic lymphocytic leukemia
CRS	Cytokine release syndrome
DDR	DNA-damage response
DNMT3A	DNA methyltransferase 3 alpha
HCT	Hematopoietic cell transplantation; autologous HCT denotes autologous hematopoietic cell transplantation
ICANS	Immune effector cell-associated neurotoxicity syndrome
MDS	Myelodysplastic neoplasm/myelodysplastic syndrome
MM	Multiple myeloma
NGS	Next-generation sequencing
NHL	Non-Hodgkin lymphoma
PARP	Poly(ADP-ribose) polymerase
PCC	Population–concept–context
PFS	Progression-free survival
PPM1D	Protein phosphatase, Mg2+/Mn2+ dependent 1D
PRISMA-ScR	Preferred Reporting Items for Systematic Reviews and Meta-Analyses Extension for Scoping Reviews
PRRT	Peptide receptor radionuclide therapy
t-AML	Therapy-related acute myeloid leukemia
t-CH	Therapy-related clonal hematopoiesis
t-MDS	Therapy-related myelodysplastic neoplasm/myelodysplastic syndrome
t-MN	Therapy-related myeloid neoplasm
TET2	Tet methylcytosine dioxygenase 2
TP53	Tumor protein p53
VAF	Variant allele frequency
WES	Whole-exome sequencing
WGS	Whole-genome sequencing
