# Supplementary material for: Clonal Hematopoiesis Across the Cancer Treatment Continuum: A Scoping Review of Clonal Selection, Evolution, and Clinical Outcomes
Source: Cancers (Basel). 2026 Oct 21;18(20):3398. doi: 10.3390/cancers18203398 (PMC13702734; doi:10.3390/cancers18203398)
Supplement: Supplementary file 1 [file cancers-18-03398-s001.zip › Supplementary File S1.pdf]

Supplementary File S1

Supplementary Tables S1–S5

Table S1 Database search strategies

Source	Search strategy / search terms
PubMed/MEDLINE	("clonal hematopoiesis" OR "clonal haematopoiesis" OR CHIP OR "clonal hematopoiesis of indeterminate potential" OR "therapy-related clonal hematopoiesis" OR "treatment-related clonal hematopoiesis" OR "therapy-selected clonal hematopoiesis") AND (chemotherapy OR radiotherapy OR radiation OR "cancer therapy" OR "cytotoxic therapy" OR "PARP inhibitor" OR "CAR-T" OR "hematopoietic stem cell transplantation" OR "autologous transplant") AND ("clonal evolution" OR "clone expansion" OR "variant allele frequency" OR VAF OR mutation OR "therapy-related myeloid neoplasm" OR "therapy-related AML" OR "therapy-related MDS")
Embase	clonal hematopoiesis/haematopoiesis terms combined with chemotherapy, radiotherapy, PARP inhibitor, CAR-T, stem cell transplantation, clonal evolution, VAF, mutation, and therapy-related myeloid neoplasm terms.
Scopus	TITLE-ABS-KEY clonal hematopoiesis/CHIP/t-CH terms AND therapy exposure terms AND clone dynamics or therapy-related myeloid neoplasm terms.
Web of Science Core Collection	Topic search using clonal hematopoiesis/CHIP/t-CH terms, therapy exposure terms, and clonal evolution/outcome terms.
Cochrane Library	Clonal hematopoiesis and cancer therapy terms.
Google Scholar	Key phrase searches for therapy-related clonal hematopoiesis, therapy-selected clonal hematopoiesis, TP53, PPM1D, PARP inhibitor, CAR-T, transplant, PRRT, and t-MN.
Citation searching	Reference lists of included original studies and major reviews were checked.

Table S2 Population concept context and eligibility criteria

Domain	Operational criterion	Reviewer note
Population	Patients with cancer, patients treated for cancer, cancer survivors, transplant recipients, and cellular-therapy recipients. Adults and children were eligible but interpreted separately when mechanisms differed.	Exclude general population CH without cancer therapy context.
Concept	Somatic clonal hematopoiesis, CHIP, CCUS, therapy-related CH, therapy-selected CH, VAF dynamics, clone expansion, new post-treatment detection, or t-MN with antecedent/concurrent CH data.	Do not assume newly detected equals newly induced unless baseline assay sensitivity supports that inference.
Context	Chemotherapy, radiotherapy, chemoradiation, PARP inhibitors, PRRT, venetoclax, lenalidomide, transplant, CAR-T, immunotherapy, survivorship, and cardio-oncology.	Attribution must account for cumulative prior therapy.
Primary evidence sources	Original human studies with extractable information on genes, VAF threshold, sample timing, treatment exposure, clonal dynamics, or outcomes.	Included in primary evidence map.
Background sources	Reviews, definitions, conceptual papers, and relevant meta-analyses used for terminology, framework building, and citation tracking.	Not counted as original extraction studies.
Main exclusions	General-population CH without a cancer-treatment context; no extractable somatic CH data; germline-only studies; animal or in vitro-only studies; conference abstracts or preprints without eligible full text; reviews or other non-primary sources; overlapping cohorts without a distinct analysis; non-informative case reports or very small series.	Reasons for exclusion recorded in the screening log.

Table S3 Source classification and evidence flow

Ref.	Short citation	Source category	Charted in primary map	Pathway	Role
[1]	Steensma et al., 2015	Contextual source	No	Outside primary map	Definitions, background or interpretation
[2]	Genovese et al., 2014	Contextual source	No	Outside primary map	Definitions, background or interpretation
[3]	Jaiswal et al., 2014	Contextual source	No	Outside primary map	Definitions, background or interpretation
[4]	Xie et al., 2014	Contextual source	No	Outside primary map	Definitions, background or interpretation
[5]	Jaiswal et al., 2019	Contextual source	No	Outside primary map	Definitions, background or interpretation
[6]	Coombs et al., 2017	Original human report	Yes	Original searches	Primary evidence mapping
[7]	Takahashi et al., 2017	Original human report	Yes	Original searches	Primary evidence mapping
[8]	Gillis et al., 2017	Original human report	Yes	Original searches	Primary evidence mapping
[9]	Hsu et al., 2018	Original human report	Yes	Original searches	Primary evidence mapping
[10]	Bolton et al., 2020	Original human report	Yes	Original searches	Primary evidence mapping
[11]	Wong et al., 2015	Original human report	Yes	Original searches	Primary evidence mapping
[12]	Coorens et al., 2021	Original human report	Yes	Original searches	Primary evidence mapping
[13]	Bertrums et al., 2022	Original human report	Yes	Original searches	Primary evidence mapping
[14]	Soerensen et al., 2020	Original human report	Yes	Original searches	Primary evidence mapping
[15]	Awada et al., 2024	Original human report	Yes	Original searches	Primary evidence mapping
[16]	Yan et al., 2024	Original human report	Yes	Original searches	Primary evidence mapping
[17]	Nead et al., 2024	Original human report	Yes	Original searches	Primary evidence mapping
[18]	Takahashi et al., 2024	Contextual source	No	Outside primary map	Definitions, background or interpretation
[19]	Kwan et al., 2021	Original human report	Yes	Original searches	Primary evidence mapping
[20]	Navitski et al., 2021	Original human report	Yes	Original searches	Primary evidence mapping
[21]	Oliveira et al., 2022	Original human report	Yes	Original searches	Primary evidence mapping
[22]	Park et al., 2020	Contextual source	No	Outside primary map	Definitions, background or interpretation
[23]	Gurnari et al., 2021	Contextual source	No	Outside primary map	Definitions, background or interpretation

[24]	Reed et al., 2023	Contextual source	No	Outside primary map	Definitions, background or interpretation
[25]	Jahn et al., 2023	Contextual source	No	Outside primary map	Definitions, background or interpretation
[26]	Sperling et al., 2017	Contextual source	No	Outside primary map	Definitions, background or interpretation
[27]	Mouhieddine et al., 2020	Original human report	Yes	Original searches	Primary evidence mapping
[28]	Singh et al., 2022	Original human report	Yes	Original searches	Primary evidence mapping
[29]	Gibson et al., 2023	Original human report	Yes	Original searches	Primary evidence mapping
[30]	Mayerhofer et al., 2023	Original human report	Yes	Original searches	Primary evidence mapping
[31]	Arends et al., 2024	Original human report	Yes	Revision supplementation	Primary evidence mapping
[32]	Morganti et al., 2024	Original human report	Yes	Original searches	Primary evidence mapping
[33]	Shyr et al., 2026	Original human report	Yes	Original searches	Primary evidence mapping
[34]	Miller et al., 2021	Original human report	Yes	Original searches	Primary evidence mapping
[35]	Blombery et al., 2022	Original human report	Yes	Original searches	Primary evidence mapping
[36]	Saini et al., 2022	Original human report	Yes	Original searches	Primary evidence mapping
[37]	Sperling et al., 2022	Original human report	Yes	Original searches	Primary evidence mapping
[38]	Teipel et al., 2022	Original human report	Yes	Original searches	Primary evidence mapping
[39]	Panagiota et al., 2023	Original human report	Yes	Original searches	Primary evidence mapping
[40]	Goldsmith et al., 2024	Original human report	Yes	Original searches	Primary evidence mapping
[41]	Avigan et al., 2025	Original human report	Yes	Original searches	Primary evidence mapping
[42]	Gustine et al., 2025	Original human report	Yes	Original searches	Primary evidence mapping
[43]	Al-Sawaf et al., 2026	Original human report	Yes	Revision supplementation	Primary evidence mapping
[44]	Rappa et al., 2026	Original human report	Yes	Revision supplementation	Primary evidence mapping
[45]	Waldschmidt et al., 2026	Original human report	Yes	Revision supplementation	Primary evidence mapping
[46]	Yoshida et al., 2022	Contextual source	No	Outside primary map	Definitions, background or interpretation
[47]	Sano et al., 2021	Experimental contextual source	No	Outside primary map	Definitions, background or interpretation
[48]	Fullin et al., 2026	Experimental contextual source	No	Outside primary map	Definitions, background or interpretation
[49]	Jaiswal et al., 2017	Contextual source	No	Outside primary map	Definitions, background or interpretation

[50]	Yura et al., 2020	Contextual source	No	Outside primary map	Definitions, background or interpretation
[51]	Saiki et al., 2021	Contextual source	No	Outside primary map	Definitions, background or interpretation
[52]	Gao et al., 2021	Contextual source	No	Outside primary map	Definitions, background or interpretation
[53]	Gu et al., 2023	Contextual source	No	Outside primary map	Definitions, background or interpretation
[54]	Rondeau et al., 2026	Contextual source	No	Outside primary map	Definitions, background or interpretation
[55]	Vanner et al., 2026	Contextual source	No	Outside primary map	Definitions, background or interpretation
[56]	Croitoru et al., 2026	Contextual source	No	Outside primary map	Definitions, background or interpretation

Table S4 PRISMA ScR reporting checklist

Item	PRISMA-ScR requirement	Reported	Location
1	Identify the report as a scoping review	Yes	Title
2	Structured summary	Yes	Abstract
3	Rationale	Yes	1.2
4	Objectives/questions	Yes	1.3
5	Protocol/registration	Yes	2.1
6	Eligibility criteria	Yes	2.2
7	Information sources	Yes	2.3
8	Search strategy	Yes	2.4; Table S1
9	Selection process	Yes	2.5
10	Data-charting process	Yes	2.6
11	Data items	Yes	2.6
12	Critical appraisal, if done	Not performed	2.8
13	Synthesis methods	Yes	2.7
14	Selection results	Yes	3.1; Figure 1
15	Source characteristics	Yes	Sections 3.2–3.3; Table 1A–E; Table S5
16	Critical appraisal results	Formal appraisal not performed; descriptive mapping provided	Sections 2.8 and 3.3; Table S5
17	Results of individual sources	Yes	Sections 3.4–3.7; Tables 1A–B, 2 and 3
18	Synthesis of results	Yes	Sections 3.2–3.8; Tables 1–3
19	Summary of evidence	Yes	4.1
20	Limitations	Yes	4.5
21	Conclusions	Yes	Section 5
22	Funding	Yes	Funding statement

Table S5 Descriptive study level mapping of methodological limitations

Study	Sampling and temporal inference	Measurement and variant attribution	Interpretive limitation
Wong et al., 2015 [11]	Antecedent and t-AML samples<br>Temporal inference: Strong	WES with targeted validation<br>VAF threshold: Assay-specific threshold<br>Variant filtering: Paired-sample somatic validation	Serial evidence supports timing; treatment attribution still depends on baseline sensitivity and alternative exposures. Reported filtering does not constitute an overall bias assessment.
Coombs et al., 2017 [6]	Single blood sample in relation to tumor profiling<br>Temporal inference: Limited	MSK-IMPACT blood–tumor paired analysis<br>VAF threshold: Not reported<br>Variant filtering: Matched tumor comparison performed; residual tumor/germline misclassification remained possible	Sampling does not establish treatment-induced or treatment-selected CH. The charted VAF threshold is unspecified. Reported filtering does not constitute an overall bias assessment.
Gillis et al., 2017 [8]	Sequential samples<br>Temporal inference: Moderate	Targeted sequencing and WES<br>VAF threshold: Not reported<br>Variant filtering: Not reported; germline classification was uncertain for some genes	Partial longitudinal or comparative evidence limits attribution to the specific therapy. The charted VAF threshold is unspecified. Variant-attribution safeguards are incompletely specified in the charting.
Takahashi et al., 2017 [7]	Pretreatment or pre-t-MN samples<br>Temporal inference: Moderate	Targeted sequencing<br>VAF threshold: Not reported<br>Variant filtering: Not reported	Partial longitudinal or comparative evidence limits attribution to the specific therapy. The charted VAF threshold is unspecified. Variant-attribution safeguards are incompletely specified in the charting.
Hsu et al., 2018 [9]	Clinical samples with functional experiments<br>Temporal inference: Limited	Clinical mutation analysis plus cell and mouse assays<br>VAF threshold: Not reported<br>Variant filtering: Not reported	Sampling does not establish treatment-induced or treatment-selected CH. The charted VAF threshold is unspecified. Variant-attribution safeguards are incompletely specified in the charting.
Bolton et al., 2020 [10]	Baseline and follow-up samples in a subset<br>Temporal inference: Strong	MSK-IMPACT CH calling and t-MN modeling<br>VAF threshold: Assay-specific threshold<br>Variant filtering: Matched tumor comparison and study-defined bioinformatic filtering	Serial evidence supports timing; treatment attribution still depends on baseline sensitivity and alternative exposures. Reported filtering does not constitute an overall bias assessment.

Mouhieddine et al., 2020 [27]	Stem-cell product or blood before/around autologous HCT<br>Temporal inference: Moderate	Targeted sequencing<br>VAF threshold: Not reported<br>Variant filtering: Not reported	Partial longitudinal or comparative evidence limits attribution to the specific therapy. The charted VAF threshold is unspecified. Variant-attribution safeguards are incompletely specified in the charting.
Soerensen et al., 2020 [14]	Leukapheresis product before autologous HCT with clinical follow-up<br>Temporal inference: Moderate	NGS plus immunophenotyping<br>VAF threshold: Not reported<br>Variant filtering: Not reported	Partial longitudinal or comparative evidence limits attribution to the specific therapy. The charted VAF threshold is unspecified. Variant-attribution safeguards are incompletely specified in the charting.
Coorens et al., 2021 [12]	Serial samples<br>Temporal inference: Strong	WGS plus deep targeted sequencing<br>VAF threshold: Assay-specific threshold<br>Variant filtering: Matched-sample analysis; detailed filtering criteria not reported	Serial evidence supports timing; treatment attribution still depends on baseline sensitivity and alternative exposures. Variant-attribution safeguards are incompletely specified in the charting.
Kwan et al., 2021 [19]	Baseline/pre-rucaparib blood<br>Temporal inference: Moderate	Targeted blood sequencing<br>VAF threshold: Not reported<br>Variant filtering: TP53 tumor/germline distinction assessed; detailed filtering method not reported	Partial longitudinal or comparative evidence limits attribution to the specific therapy. The charted VAF threshold is unspecified. Variant-attribution safeguards are incompletely specified in the charting.
Miller et al., 2021 [34]	Pre-infusion CH assessment with post-infusion outcomes<br>Temporal inference: Limited	Targeted blood sequencing<br>VAF threshold: Not reported<br>Variant filtering: Not reported	Sampling does not establish treatment-induced or treatment-selected CH. The charted VAF threshold is unspecified. Variant-attribution safeguards are incompletely specified in the charting.
Navitski et al., 2021 [20]	Before PARP-inhibitor decision; blood and/or marrow<br>Temporal inference: Limited	NGS<br>VAF threshold: Not reported<br>Variant filtering: Germline context considered; somatic-filtering method not reported	Sampling does not establish treatment-induced or treatment-selected CH. The charted VAF threshold is unspecified. Variant-attribution safeguards are incompletely specified in the charting.
Bertrums et al., 2022 [13]	Paired/serial samples in relation to therapy and t-MN<br>Temporal inference: Strong	WGS with mutational-age/signature analysis<br>VAF threshold: Assay-specific threshold<br>Variant filtering: Matched-sample and	Serial evidence supports timing; treatment attribution still depends on baseline sensitivity and alternative exposures.

		mutational-signature analysis	Reported filtering does not constitute an overall bias assessment.
Blombery et al., 2022 [35]	Baseline and serial treatment samples<br>Temporal inference: Moderate	Targeted sequencing<br>VAF threshold: Not reported<br>Variant filtering: Not reported	Partial longitudinal or comparative evidence limits attribution to the specific therapy. The charted VAF threshold is unspecified. Variant-attribution safeguards are incompletely specified in the charting.
Oliveira et al., 2022 [21]	At MDS/AML evaluation<br>Temporal inference: Limited	Marrow morphology, cytogenetics and NGS<br>VAF threshold: Not reported<br>Variant filtering: Somatic versus germline classification was uncertain	Sampling does not establish treatment-induced or treatment-selected CH. The charted VAF threshold is unspecified. Reported filtering does not constitute an overall bias assessment.
Saini et al., 2022 [36]	Pre-infusion CH assessment with post-infusion outcomes<br>Temporal inference: Limited	Targeted blood sequencing<br>VAF threshold: Not reported<br>Variant filtering: Tumor-origin review reported; detailed filtering criteria not reported	Sampling does not establish treatment-induced or treatment-selected CH. The charted VAF threshold is unspecified. Variant-attribution safeguards are incompletely specified in the charting.
Singh et al., 2022 [28]	Baseline and serial follow-up samples<br>Temporal inference: Moderate	100-gene targeted panel<br>VAF threshold: VAF ≥1%<br>Variant filtering: Not reported	Partial longitudinal or comparative evidence limits attribution to the specific therapy. Variant-attribution safeguards are incompletely specified in the charting.
Sperling et al., 2022 [37]	Clinical specimens plus functional experiments<br>Temporal inference: Moderate	Genomic and functional analyses<br>VAF threshold: Not reported<br>Variant filtering: Not reported	Partial longitudinal or comparative evidence limits attribution to the specific therapy. The charted VAF threshold is unspecified. Variant-attribution safeguards are incompletely specified in the charting.
Teipel et al., 2022 [38]	Samples collected around CAR-T treatment<br>Temporal inference: Moderate	Targeted sequencing<br>VAF threshold: Study-defined CHIP threshold<br>Variant filtering: Not reported	Partial longitudinal or comparative evidence limits attribution to the specific therapy. Variant-attribution safeguards are incompletely specified in the charting.
Gibson et al., 2023 [29]	Longitudinal blood samples<br>Temporal inference: Strong	Targeted sequencing<br>VAF threshold: VAF ≥2%<br>Variant filtering: Population-frequency/bioinformatic filtering; detailed criteria not reported	Serial evidence supports timing; treatment attribution still depends on baseline sensitivity and alternative exposures.

			Variant-attribution safeguards are incompletely specified in the charting.
Mayerhofer et al., 2023 [30]	Baseline, 6 weeks and 12 weeks<br>Temporal inference: Strong	Targeted sequencing<br>VAF threshold: Not reported<br>Variant filtering: Population-frequency/bioinformatic filtering; detailed criteria not reported	Serial evidence supports timing; treatment attribution still depends on baseline sensitivity and alternative exposures. The charted VAF threshold is unspecified. Variant-attribution safeguards are incompletely specified in the charting.
Panagiota et al., 2023 [39]	Pre-infusion and serial post-infusion samples<br>Temporal inference: Strong	Error-corrected targeted sequencing<br>VAF threshold: VAF ≥1%<br>Variant filtering: Error-corrected sequencing; detailed germline/tumor filtering not reported	Serial evidence supports timing; treatment attribution still depends on baseline sensitivity and alternative exposures. Variant-attribution safeguards are incompletely specified in the charting.
Arends et al., 2024 [27]	Baseline and serial on-treatment whole blood/plasma<br>Temporal inference: Strong	Error-corrected 72-gene panel; selected single-cell analyses<br>VAF threshold: CH VAF ≥1%; calling floor 0.1%; serial retention ≥0.8%<br>Variant attribution: VAF/population-frequency germline filtering; paired blood/cfDNA attribution; selected single-cell validation	Serial blood/plasma assays support clone timing; combined therapies and prior exposure complicate drug-specific attribution.
Awada et al., 2024 [31]	Pre-HCT and follow-up/diagnostic samples<br>Temporal inference: Strong	Serial marrow molecular profiling<br>VAF threshold: Not reported<br>Variant filtering: Not reported	Serial evidence supports timing; treatment attribution still depends on baseline sensitivity and alternative exposures. The charted VAF threshold is unspecified. Variant-attribution safeguards are incompletely specified in the charting.
Goldsmith et al., 2024 [40]	Pre-infusion assessment<br>Temporal inference: Limited	108-gene targeted exome sequencing (~1000×)<br>VAF threshold: VAF ≥2%<br>Variant filtering: Not reported	Sampling does not establish treatment-induced or treatment-selected CH. Variant-attribution safeguards are incompletely specified in the charting.
Morganti et al., 2024 [32]	Baseline and longitudinal follow-up<br>Temporal inference: Strong	Error-corrected targeted sequencing<br>VAF threshold: VAF ≥2%; exploratory threshold ≥0.5%<br>Variant filtering: Error-corrected bioinformatic filtering; detailed germline/tumor criteria not reported	Serial evidence supports timing; treatment attribution still depends on baseline sensitivity and alternative exposures. Variant-attribution safeguards are incompletely specified in the charting.

Nead et al., 2024 [17]	Before and after chemoradiation<br>Temporal inference: Strong	Error-corrected duplex sequencing<br>VAF threshold: Assay-specific threshold<br>Variant filtering: Duplex error correction; detailed germline/tumor filtering not reported	Serial evidence supports timing; treatment attribution still depends on baseline sensitivity and alternative exposures. Variant-attribution safeguards are incompletely specified in the charting.
Yan et al., 2024 [16]	Pre-autologous HCT material with longitudinal follow-up<br>Temporal inference: Moderate	Targeted sequencing<br>VAF threshold: Not reported<br>Variant filtering: Not reported	Partial longitudinal or comparative evidence limits attribution to the specific therapy. The charted VAF threshold is unspecified. Variant-attribution safeguards are incompletely specified in the charting.
Avigan et al., 2025 [41]	Pre-infusion CH assessment with post-infusion outcomes<br>Temporal inference: Limited	CH testing plus inflammatory markers<br>VAF threshold: Not reported<br>Variant filtering: Not reported	Sampling does not establish treatment-induced or treatment-selected CH. The charted VAF threshold is unspecified. Variant-attribution safeguards are incompletely specified in the charting.
Gustine et al., 2025 [42]	Pre-infusion marrow with outcome follow-up<br>Temporal inference: Limited	Targeted exome sequencing of marrow<br>VAF threshold: VAF ≥2%<br>Variant filtering: Tumor-derived TP53 variants excluded when origin was uncertain	Sampling does not establish treatment-induced or treatment-selected CH. Reported filtering does not constitute an overall bias assessment.
Al-Sawaf et al., 2026 [43]	CLL12 baseline; CLL14 partial baseline and serial treatment/follow-up samples<br>Temporal inference: Moderate	Error-corrected targeted sequencing; selected WES/single-cell analyses<br>VAF threshold: VAF ≥0.5%; CHIP also summarized at ≥2%<br>Variant attribution: CD19-negative sorted cells in CLL12; MRD-informed attribution in CLL14; selected single-cell validation	Serial sampling is incomplete or confined to a selected subset; prior exposure, selection and measurement limits constrain causal attribution.
Rappa et al., 2026 [44]	Around lymphodepletion; serial post-infusion PBMCs in a subset<br>Temporal inference: Moderate	Conventional 74-gene targeted panel; serum proteomics<br>VAF threshold: Variants VAF ≥1%; proportions also reported at ≥2%<br>Variant attribution: Flow cytometry excluded circulating malignancy; tumor/marrow comparison showed no variant overlap; germline strategy incompletely detailed	Serial sampling is incomplete or confined to a selected subset; prior exposure, selection and measurement limits constrain causal attribution.
Shyr et al., 2026 [33]	Biobank sample with longitudinal cardiovascular follow-up<br>Temporal inference: Limited	Whole-genome sequencing; 58-gene CHIP definition<br>VAF threshold: VAF ≥2%<br>Variant filtering: Population-frequency/bioinformatic germline filtering; detailed criteria not reported	Sampling does not establish treatment-induced or treatment-selected CH. Variant-attribution safeguards are incompletely specified in the charting.

Waldschmidt et al., 2026 [45]	Pretreatment material available in 8/12 cases; serial CD138-negative marrow in 10/12<br>Temporal inference: Moderate	WGS in selected cases/controls plus informed targeted sequencing<br>VAF threshold: Driver mutation VAF ≥2% for CCUS; separate analytical LoD not reported<br>Variant attribution: CD138-negative marrow; WGS/melphalan-signature analysis; comprehensive germline filtering not detailed in the letter	Serial sampling is incomplete or confined to a selected subset; prior exposure, selection and measurement limits constrain causal attribution.

Abbreviations: AML, acute myeloid leukemia; CAR-T, chimeric antigen receptor T-cell therapy; CH, clonal hematopoiesis; CHIP, clonal hematopoiesis of indeterminate potential; HCT, hematopoietic cell transplantation; MDS, myelodysplastic neoplasm / myelodysplastic syndrome; NGS, next-generation sequencing; TP53, tumor protein p53; VAF, variant allele frequency; WES, whole-exome sequencing; WGS, whole-genome sequencing; t-AML, therapy-related acute myeloid leukemia; t-MN, therapy-related myeloid neoplasm; cfDNA, cell-free DNA; PBMC, peripheral blood mononuclear cells; MRD, measurable residual disease; LoD, limit of detection; U2AF1, U2 small nuclear RNA auxiliary factor 1; CD19/CD138, cluster of differentiation 19/138.
