# Supplementary material for: Clonal Hematopoiesis Across the Cancer Treatment Continuum: A Scoping Review of Clonal Selection, Evolution, and Clinical Outcomes
Source: Cancers (Basel). 2026 Oct 21;18(20):3398. doi: 10.3390/cancers18203398 (PMC13702734; doi:10.3390/cancers18203398)
Supplement: Supplementary file 1 [file cancers-18-03398-s001.zip › Supplementary File S2.pdf]

Supplementary File S2

Reports excluded after full text assessment

Log entry	Study	Reason for exclusion
1	Ohad Oren et al. (2026). Clinical and genetic landscape of individuals evaluated in a CHIP cardiology clinic: A single-center experience, 2020 to 2025. Journal of Clinical Oncology. DOI: 10.1200/jco.2026.44.16_suppl.6593	No therapy-related context
2	Xuesen Zheng et al. (2023). An Integrated Single Cell Immunogenomic Atlas Reveals Pathways of Clonal Expansion, Inflammation and Dyserythropoiesis in Low and High-Risk Clonal Haematopoiesis Blood. DOI: 10.1182/blood-2023-174947	No therapy-related context
3	David J. Curtis et al. (2022). The Growth Kinetics of Clonal Hematopoiesis Inform Risk of Blood Cancer Progression Blood. DOI: 10.1182/blood-2022-168526	No therapy-related context
4	M. Naldini et al. (2023). Longitudinal single-cell profiling of chemotherapy response in acute myeloid leukemia Nature Communications. DOI: 10.1038/s41467-023-36969-0	No therapy-related context
5	Sandra Novoa Jáuregui et al. (2024). Clonal Hematopoiesis in Relatives of Adults with Myeloid Neoplasms with Germline Predisposition Blood. DOI: 10.1182/blood-2024-209611	No therapy-related context
6	C. Swanton (2022). Abstract IA12: Cancer evolution: Chromosomal instability and immune evasion Interrogating the Immune Landscape of Cancer. DOI: 10.1158/2326-6074.tumimm21-ia12	No therapy-related context
7	E. Gumuser et al. (2022). Abstract 10097: Clinical Presentations and Outcomes of Atherosclerotic Cardiovascular Disease With and Without Underlying Clonal Hematopoiesis Circulation. DOI: 10.1161/circ.146.suppl_1.10097	No therapy-related context
8	Chang-Ying Jiang et al. (2019). Transcriptomic Heterogeneity and Clonal Evolution Associated with Therapeutic Resistance in Mantle Cell Lymphoma Revealed By Single Cell RNA-Seq Blood. DOI: 10.1182/blood-2019-129134	No therapy-related context
9	M. Uddin et al. (2022). Longitudinal profiling of clonal hematopoiesis provides insight into clonal dynamics Immunity & Ageing. DOI: 10.1186/s12979-022-00278-9	No therapy-related context
10	K. Kirschner et al. (2024). FITNESS AND TIME OF MUTATION ACQUISITION A BETTER PREDICTOR OF OUTCOME IN CLONAL HEMOPOIESIS COMPARED TO CLONE SIZE Innovation in aging. DOI: 10.1093/geroni/igae098.1448	No therapy-related context
11	Naomi Iris van den Berg et al. (2025). TRACERx as an Optimized Paradigm for Understanding Cancer Evolution Annual Review of Cancer Biology. DOI: 10.1146/annurev-cancerbio-070824-125326	No therapy-related context
12	Celine Kerros et al. (2020). Abstract 1516: Deep profiling of T-cell repertoire and tumor heterogeneity in chronic lymphocytic leukemia patients following allogeneic T-cell therapy Tumor Biology. DOI: 10.1158/1538-7445.am2020-1516	No therapy-related context
13	D. Hammond et al. (2025). A multi-site break through cancer pilot study testing the feasibility and safety of therapeutic intervention for patients with high-risk clonal cytopenia of undetermined significance (CCUS) Blood. DOI: 10.1182/blood-2025-3160	No therapy-related context
14	Chiara Motisi et al. (2026). Clonal hematopoiesis of undetermined potential as an emerging determinant of inflammageing, and age-related diseases: potential role as biomarker and target. Ageing Research Reviews. DOI: 10.1016/j.arr.2026.103296	No therapy-related context
15	Olisaemeka D. Ogbue et al. (2023). Can Emergency Hematopoiesis Due to Sepsis Lead to Clonal Hematopoiesis? Implications for Understanding the Pathogenesis of Clonal Evolution Blood. DOI: 10.1182/blood-2023-187424	No therapy-related context

16	A. Rojek et al. (2025). Real-World Outcomes of Relapsed/Refractory Core-Binding Factor Acute Myeloid Leukemia: a COMMAND Registry Study<br>American journal of hematology/oncology. DOI: 10.1002/ajh.27664	No therapy-related context
17	Soo-Jung Park et al. (2018). Clonal Hematopoiesis in Aging<br>Current Stem Cell Reports. DOI: 10.1007/s40778-018-0133-9	No therapy-related context
18	Robert Yuan et al. (2026). Modern Concepts in Precision Targeting of Acute Myeloid Leukemia Based on Principles of Molecular Oncology<br>Cancers. DOI: 10.3390/cancers18142329	No therapy-related context
19	Vishakha Kasherwal et al. (2025). Extracellular vesicles secreted by leukemic cells as mediators of dysregulated hematopoiesis: acute myeloid leukemia as a case in point<br>Expert Review of Hematology. DOI: 10.1080/17474086.2025.2471860	No therapy-related context
20	Siddhartha Jaiswal et al. (2019). Clonal hematopoiesis in human aging and disease<br>Science. DOI: 10.1126/science.aan4673 Main manuscript reference [5].	No therapy-related context
21	A. Klopp (2026). Longitudinal Profiling of Cervical Cancers Reveals Therapy-Induced Vulnerabilities Beyond the Checkpoint.<br>Cancer Research. DOI: 10.1158/0008-5472.can-25-5767	No therapy-related context
22	Olisaemeka D. Ogbue et al. (2024). Impact of Sepsis and Stress Hematopoiesis on the Acquisition and Persistence of Clonal Hematopoiesis<br>Blood. DOI: 10.1182/blood-2024-209240	No therapy-related context
23	S. Scala (2026). A09 | Defining the clonal complexity and biology of gene-modified hematopoiesis in gene therapy<br>Haematologica. DOI: 10.3324/haematol.2026.s1.9	No therapy-related context
24	Ashton A Connor et al. (2021). Pancreatic cancer evolution and heterogeneity: integrating omics and clinical data<br>Nature Reviews. Cancer. DOI: 10.1038/s41568-021-00418-1	No therapy-related context
25	A. Aleshin et al. (2018). Single-Cell Mutational Profiling of Clonal Evolution in De Novo AML during Therapy and Relapse<br>Blood. DOI: 10.1182/BLOOD-2018-99-118089	No therapy-related context
26	Hajime Okada et al. (2026). Holding the Line: Negative Selection Maintains the Karyotype in Metastatic Colorectal Cancer.<br>Cancer Discovery. DOI: 10.1158/2159-8290.CD-25-1430	No therapy-related context
27	S. Hautaniemi et al. (2024). Abstract 1617: Tumor evolution during therapy in ovarian cancer<br>Cancer Research. DOI: 10.1158/1538-7445.am2024-1617	No therapy-related context
28	A. Tall et al. (2022). Clonal hematopoiesis in cardiovascular disease and therapeutic implications<br>Nature Cardiovascular Research. DOI: 10.1038/s44161-021-00015-3	No therapy-related context
29	D. Eastburn et al. (2018). Longitudinal Monitoring of AML Tumors with High-Throughput Single-Cell DNA Sequencing Reveals Rare Clones Prognostic for Disease Progression and Therapy Response<br>Blood. DOI: 10.1182/BLOOD-2018-99-119869	No therapy-related context
30	R. Chebbi et al. (2026). Abstract 1511: Longitudinal whole genome sequencing to investigate clonal hematopoiesis dynamics<br>Cancer Research. DOI: 10.1158/1538-7445.am2026-1511	No therapy-related context
31	N. Key et al. (2020). Venous Thromboembolism Prophylaxis and Treatment in Patients With Cancer: ASCO Clinical Practice Guideline Update.<br>Journal of Clinical Oncology. DOI: 10.1200/JCO.19.01461	No therapy-related context
32	H. Dang et al. (2017). ClonEvol: clonal ordering and visualization in cancer sequencing<br>Annals of Oncology. DOI: 10.1093/annonc/mdx517	No extractable CH data
33	Raphael Hablesreiter et al. (2024). Intra-Tumor Heterogeneity in Core-Binding Factor Acute Myeloid Leukemia<br>Blood. DOI: 10.1182/blood-2024-202342	No extractable CH data
34	Jennifer J. VanOudenhove et al. (2022). Is it the time to integrate novel sequencing technologies into clinical practice?<br>Current opinion in hematology. DOI: 10.1097/MOH.0000000000000754	No extractable CH data
35	A. Garrido et al. (2020). Risk-Adapted Intensive Chemotherapy for Primary ACUTE Myeloid Leukemia during the Last 25 YEARS: Increase in Complete Remission RATE, Hematopoietic Cell Transplantation Access and Decrease in Relapse Incidence Have LED to Improved Survival<br>Blood. DOI: 10.1182/blood-2020-140680	No extractable CH data

36	W. Liao et al. (2019). Tumor and microenvironment evolution during chemotherapy combine with bevacizumab in colorectal cancer liver metastasis.<br>Journal of Clinical Oncology. DOI: 10.1200/JCO.2019.37.15_SUPPL.3568	No extractable CH data
37	P. Iyengar (2022). Local Therapy for Oligometastatic Disease-Cart Before the Horse?<br>International Journal of Radiation Oncology, Biology, Physics. DOI: 10.1016/j.ijrobp.2022.09.013	No extractable CH data
38	K. Khan et al. (2018). Longitudinal liquid biopsy and mathematical modelling of clonal evolution forecast waiting time to treatment failure in the PROSPECT-C phase II colorectal cancer clinical trial<br>Cancer Discovery. DOI: 10.1158/2159-8290.CD-17-0891	No extractable CH data
39	H. Sabaawy (2013). Genetic Heterogeneity and Clonal Evolution of Tumor Cells and their Impact on Precision Cancer Medicine<br>Journal of Leukemia. DOI: 10.4172/2329-6917.1000124	No extractable CH data
40	Shuang Meng et al. (2026). Therapy-induced immunoediting and the evolution of cancer immune escape<br>Frontiers in Immunology. DOI: 10.3389/fimmu.2026.1936590	No extractable CH data
41	John Nemunaitis et al. (2023). Clonal Neoantigen: Emerging “Mechanism-based” Biomarker of Immunotherapy Response<br>Cancers. DOI: 10.3390/cancers15235616	No extractable CH data
42	2026. Circulating Tumour DNA as Resistance Biomarkers: Real-Time Monitoring of Therapeutic Response.<br>Journal of Chemical Health Risks. DOI: 10.52783/jchr.v16.i2.13364	No extractable CH data
43	L. Welter et al. (2019). Abstract P3-01-09: Tracing clonal evolution of circulating tumor cells and cell-free DNA in a metastatic breast cancer patient<br>Poster Session Abstracts. DOI: 10.1158/1538-7445.sabcs18-p3-01-09	No extractable CH data
44	M. Andrews et al. (2017). Cancer evolution during immunotherapy<br>Cell. DOI: 10.1016/j.cell.2017.10.027	No extractable CH data
45	J. Lowder et al. (2015). Will next-generation agents deliver on the promise of epigenetic hypomethylation therapy?<br>Epigenomics. DOI: 10.2217/epi.15.66	No extractable CH data
46	Angela Todorovski et al. (2024). CHIP away at the marrow-clot connection: inflammation, clonal hematopoiesis, and thromboembolic disease<br>Blood Advances. DOI: 10.1182/bloodadvances.2024014430	Non-primary/contextual source
47	Wolfgang E. Schleicher et al. (2024). CHIP: a clonal odyssey of the bone marrow niche<br>Journal of Clinical Investigation. DOI: 10.1172/JCI180068	Non-primary/contextual source
48	Ohad Oren et al. (2026). Clonal haematopoiesis of indeterminate potential in cardiovascular medicine: is it time for selective testing?<br>European Heart Journal. DOI: 10.1093/eurheartj/ehag454	Non-primary/contextual source
49	Jose-Mario Capo-Chichi et al. (2019). Emerging patterns in clonal haematopoiesis<br>Journal of Clinical Pathology. DOI: 10.1136/jclinpath-2019-205851	Non-primary/contextual source
50	Murphy AJ, Dragoljevic D, Natarajan P, Wang N. Hematopoiesis of Indeterminate Potential and Atherothrombotic Risk. Thromb Haemost. 2022 Sep;122(9):1435-1442. doi: 10.1055/a-1830-2147. Epub 2022 Apr 20. PMID: 35445383; PMCID: PMC9420552.	Non-primary/contextual source
51	S. Reed et al. (2022). CHIP Happens: Clonal hematopoiesis of indeterminate potential and its relationship to solid tumors<br>Clinical Cancer Research. DOI: 10.1158/1078-0432.CCR-22-2598 Main manuscript reference [24].	Non-primary/contextual source
52	Kyung-Duk Min et al. (2020). The role of clonal haematopoiesis in cardiovascular diseases: epidemiology and experimental studies<br>Journal of Internal Medicine. DOI: 10.1111/joim.13130	Non-primary/contextual source
53	R. Belizaire et al. (2023). Clonal haematopoiesis and dysregulation of the immune system<br>Nature reviews. Immunology. DOI: 10.1038/s41577-023-00843-3	Non-primary/contextual source
54	Matthew Sadler et al. (2026). Clonal haematopoiesis and outcomes after acute coronary syndrome: a systematic review and meta-analysis.<br>ESC Heart Failure. DOI: 10.1093/eschf/xvag191	Non-primary/contextual source
55	Libby P, Sidlow R, Lin AE, Gupta D, Jones LW, Moslehi J, Zeiher A, Jaiswal S, Schulz C, Blankstein R, Bolton KL, Steensma D, Levine RL, Ebert BL. Clonal Hematopoiesis: Crossroads of Aging, Cardiovascular Disease, and Cancer: JACC Review Topic of the Week. J Am Coll Cardiol. 2019 Jul 30;74(4):567-577. doi:	Non-primary/contextual source

	10.1016/j.jacc.2019.06.007. PMID: 31345432; PMCID: PMC6681657.	
56	Mohammed Hasan Khan et al. (2022). Hypertension in Cancer Survivors<br>Current Hypertension Reports. DOI: 10.1007/s11906-022-01208-2	Non-primary/contextual source
57	J. B. Menendez-Gonzalez et al. (2022). Exploring the Associations Between Clonal Hematopoiesis of Indeterminate Potential, Myeloid Malignancy, and Atherosclerosis.<br>Methods in molecular biology. DOI: 10.1007/978-1-0716-1924-7_5	Non-primary/contextual source
58	Kenji Harada et al. (2026). Patient-derived organoids for modeling therapy tolerance, persistence, and resistance in cancer<br>Molecular Cancer. DOI: 10.1186/s12943-026-02800-9	Non-primary/contextual source
59	2021. Cancer Science: In this Issue<br>Cancer Science. DOI: 10.1111/cas.15135	Non-primary/contextual source
60	C. Dinardo et al. (2019). Acute Myeloid Leukemia: from Mutation Profiling to Treatment Decisions<br>Current Hematologic Malignancy Reports. DOI: 10.1007/s11899-019-00535-7	Non-primary/contextual source
61	C. Taborda et al. (2024). Clonal cytopenia of undetermined significance: definitions, risk and therapeutic targets<br>Frontiers in Hematology. DOI: 10.3389/frhem.2024.1419323	Non-primary/contextual source
62	Lakshmi Thulluri et al. (2026). Clonal hematopoiesis and cardiovascular outcomes after hematopoietic cell transplantation: A systematic review and meta-analysis.<br>Journal of Clinical Oncology. DOI: 10.1200/jco.2026.44.16_suppl.e18578	Non-primary/contextual source
63	Xuejiao Zhang et al. (2026). Clonal Hematopoiesis of Indeterminate Potential in Cardiovascular Disease: Gene-Specific Mechanisms and Therapeutic Implications<br>International Journal of General Medicine. DOI: 10.2147/IJGM.S626604	Non-primary/contextual source
64	A. Ørskov et al. (2017). DNA Methyltransferase Inhibitors in Myeloid Cancer: Clonal Eradication or Clonal Differentiation?<br>Cancer Journal. DOI: 10.1097/PPO.0000000000000282	Non-primary/contextual source
65	J. Martignoles et al. (2018). Genetic Hierarchy of Acute Myeloid Leukemia: From Clonal Hematopoiesis to Molecular Residual Disease<br>International Journal of Molecular Sciences. DOI: 10.3390/ijms19123850	Non-primary/contextual source
66	Chunmei Ye et al. (2026). The eco-evolutionary dynamics of acute myeloid leukemia: From clonal games to clinical intervention.<br>Cancer Treatment and Research Communications. DOI: 10.1016/j.ctarc.2026.101349	Non-primary/contextual source
67	O. Calvillo-Arguelles et al. (2019). Connections Between Clonal Hematopoiesis, Cardiovascular Disease, and Cancer: A Review<br>JAMA cardiology. DOI: 10.1001/jamacardio.2019.0302	Non-primary/contextual source
68	David M. Swoboda et al. (2019). Mutation-Driven Therapy in MDS<br>Current Hematologic Malignancy Reports. DOI: 10.1007/s11899-019-00554-4	Non-primary/contextual source
69	H. Qin et al. (2022). Effects of isocitrate dehydrogenase mutations on treatment outcomes in patients with acute myeloid leukemia: a systematic review and meta-analysis<br>. DOI: 10.37766/inplasy2022.9.0008	Non-primary/contextual source
70	Bing Li et al. (2018). Leukemic Transformation of Myeloproliferative Neoplasms: Therapeutic and Genomic Considerations<br>Current Hematologic Malignancy Reports. DOI: 10.1007/s11899-018-0491-5	Non-primary/contextual source
71	V. Pinnelli et al. (2026). Three generations of targeted therapies in chronic myeloid leukemia: Molecular advances, adverse effects, therapeutic challenges, and future perspectives<br>Eurasian Journal of Medicine and Oncology. DOI: 10.36922/ejmo026270318	Non-primary/contextual source
72	K. Maher et al. (2025). Toward a Deeper Understanding of Clonal Evolution in Acute Myeloid Leukemia: Translational and Clinical Impacts<br>Journal of Cellular Signaling. DOI: 10.33696/signaling.6.133	Non-primary/contextual source
73	N. Beerenwinkel et al. (2014). Cancer Evolution: Mathematical Models and Computational Inference<br>Systematic Biology. DOI: 10.1093/sysbio/syu081	Non-primary/contextual source
74	Ryan M. Carr et al. (2019). Genetic and epigenetic factors interacting with clonal hematopoiesis resulting in chronic myelomonocytic leukemia.<br>Current opinion in hematology. DOI: 10.1097/MOH.0000000000000553	Non-primary/contextual source

75	Monaco F et al. (2026). Case Report: donor cell-derived leukemia after allogeneic stem cell transplantation for metastatic renal cell carcinoma<br>Frontiers in Immunology, 17:1830323. DOI: 10.3389/fimmu.2026.1830323	Non-informative case report or small series
76	Aldoss I et al. (2020). Multiple donor-derived leukemias in a recipient of allogeneic hematopoietic cell transplantation for myeloid malignancy<br>Blood Advances, 4(19):4798–4801. DOI: 10.1182/bloodadvances.2020002803	Non-informative case report or small series
77	Plesa A et al. (2022). Donor-Derived Leukemia in a Recipient of Double-Unit Cord Blood Transplantation for Acute Myeloid Leukemia: A Case Study and Literature Review<br>Oncology and Therapy, 10:75–84. DOI: 10.1007/s40487-021-00180-2	Non-informative case report or small series
78	An R et al. (2025). Therapy related acute myeloid leukemia secondary to CAR-T therapy in refractory/relapsed multiple myeloma patient showing poor prognosis<br>Annals of Hematology, 104:2013–2016. DOI: 10.1007/s00277-025-06310-4	Non-informative case report or small series
79	Mroczkowska-Bękarciak A et al. (2025). A case report of donor cell–derived hematologic neoplasms 9 years after allogeneic hematopoietic cell transplantation<br>Oncotarget, 16:44–50. DOI: 10.18632/oncotarget.28686	Non-informative case report or small series
80	Y. Pershad et al. (2025). Correlates and Consequences of Clonal Hematopoiesis Expansion Rate: A 15-Year Longitudinal Study of 6,986 Women<br>medRxiv. DOI: 10.1101/2025.01.12.25320422	Abstract/preprint only
81	Andrew W. McPherson et al. (2018). Observing Clonal Dynamics across Spatiotemporal Axes: A Prelude to Quantitative Fitness Models for Cancer.<br>Cold Spring Harbor Perspectives in Medicine. DOI: 10.1101/cshperspect.a029603	Abstract/preprint only
82	C. Engen et al. (2020). Converging molecular evolution in acute myeloid leukaemia<br>medRxiv. DOI: 10.1101/2020.11.03.20222885	Abstract/preprint only
83	F. Thomas et al. (2017). Cancer adaptations: Atavism, de novo selection, or something in between?<br>Bioessays. DOI: 10.1002/bies.201700039	Non-human study
84	Bailey Kane et al. (2025). Abstract B012: A review of experimental evidence for cost of therapeutic resistance in cancer<br>Cancer Research. DOI: 10.1158/1538-7445.canevol25-b012	Non-human study
85	Goldsmith SR et al. (2023). Clonal Hematopoiesis Is Associated with Severe Cytokine Release Syndrome in Patients Treated with Chimeric Antigen Receptor T-Cell (CAR-T) Therapy<br>Blood, 142(Supplement 1), abstract 4814. DOI: 10.1182/blood-2023-185157	Overlapping cohort without distinct analysis
86	Singh A et al. (2020). Clonal Hematopoiesis in Patients with Neuroendocrine Tumors Receiving Peptide Receptor Radionuclide Therapy (PRRT)<br>Blood, 136(Supplement 1):35, abstract 1844. DOI: 10.1182/blood-2020-141570	Overlapping cohort without distinct analysis
