# Supplementary material for: Clonal Hematopoiesis Across the Cancer Treatment Continuum: A Scoping Review of Clonal Selection, Evolution, and Clinical Outcomes
Source: Cancers (Basel). 2026 Oct 21;18(20):3398. doi: 10.3390/cancers18203398 (PMC13702734; doi:10.3390/cancers18203398)
Supplement: Supplementary file 1 [file cancers-18-03398-s001.zip › Supplementary File S3.pdf]

File S3 Study data extraction form

Not intended for publication

**Field**	**Extracted information**	**Source page or table**
**A Study identification and source classification**		
Study ID and shared cohort ID		
Full citation and publication year		
Article title; DOI; PMID; study registration if reported		
Country, center(s), recruitment dates and funding/conflicts		
Source category: original human / contextual / experimental		
Evidence source/pathway and search or update date		
Design: cohort / case–control / case series / clinical-mechanistic or genomic / other		
Cancer type, disease stage and treatment status		
Eligibility decision and reason; linked exclusion record if excluded		
Overlapping publications and distinct contribution		
Extractor name, extraction date and verification/recheck date		
Language; peer-review and full-text availability		
**B Population and treatment exposure**		
Total participants; sequenced subset; paired-sample subset		
CH-positive and CH-negative denominators; exclusions		
Age summary and range; pediatric / adult / mixed		
Sex and relevant baseline clinical characteristics		
Baseline blood counts, cytopenia and marrow involvement		
Inherited cancer predisposition and germline context		
Primary therapy/context used for mutually exclusive study counts		
Specific drug(s), combinations, modality and dose where reported		
Treatment duration, cycles and cumulative exposure		
Prior therapy lines, agents, radiotherapy and time since exposure		
Transplantation, conditioning, mobilization and maintenance		
CAR-T target/product, lymphodepletion and infusion date/time anchor		
Exposure comparison group and relevant co-interventions		
Exposure attribution limits and prior-treatment confounding		

**C Sampling sequencing and variant filtering**		
Specimen: peripheral blood / marrow / stem-cell product / other		
Cell fraction, sorted lineage, PBMC or bulk material		
Pretreatment/baseline sampling in relation to exposure		
On-treatment and post-treatment sampling times		
Number of timepoints; sampling interval; follow-up duration		
Sequencing platform; panel/exome/WGS; gene list/panel size		
Coverage/depth and molecular depth where reported		
Error correction, unique molecular identifiers or duplex methods		
Assay limit of detection and validation details		
Variant-calling threshold and separate retention/reporting threshold		
Study CH/CHIP/CCUS definition and VAF criterion		
Matched nonhematopoietic sample and germline filtering		
Matched tumor review and circulating-tumor contamination checks		
Population-frequency, copy-number and allele-balance handling		
Orthogonal or lineage-specific confirmation; missing methods		
Threshold guidance	Record calling sensitivity and the final analysis threshold separately.	
**D Clone findings and temporal interpretation**		
Recording instructions	Record each clone/variant separately. Link variants only when lineage or single-cell evidence supports a shared clone.	
Clone/variant ID; gene/pathway; HGVS or reported alteration		
Baseline VAF and sampling time; detection/absence evidence		
Follow-up VAF(s), times and absolute or relative changes		
VAF uncertainty, copy-number effects and cell-composition changes		
Co-mutations and whether clonal co-occurrence is demonstrated		
Cytogenetics/mosaic chromosomal alterations; 5q/7q or complex karyotype		
Clonal fitness model, estimates, units and growth classification		
Mutation-signature method and treatment-associated signatures		
Clonal continuity to t-MN: samples, lineage and supporting evidence		
Trajectory state(s), time interval and supporting evidence	Choose: pre-existing selection/expansion; newly detected with induction uncertain; de novo induction supported; stable/persistent; contraction/disappearance; progression to t-MN; indeterminate. Record multiple states where applicable.	

Temporal inference category and justification	Strong: comparable sensitive baseline/follow-up samples. Moderate: partial serial samples or credible comparison group. Limited: cross-sectional or post-treatment only. Unclear/not assessable: insufficient timing or assay information.	
Selection, induction or outcome modification and supporting evidence		
Interpretation guidance	Temporal categories are not risk-of-bias or certainty ratings. Post-treatment detection alone does not establish induction; record baseline sensitivity and alternative explanations.	
**E Clinical outcomes and effect estimates**		
Recording instructions	Record each endpoint and population/subgroup separately; preserve its definition, denominator, time origin and model.	
Outcome domain and exact definition/grade		
Population/subgroup and number analyzed		
Treatment/sampling time origin and follow-up duration		
Event counts, denominators and competing events		
Exposure/comparator: CH status, genotype, clone size or trajectory		
Effect measure: HR / OR / RR / difference / other		
Effect estimate, 95% CI and p value as reported		
Adjusted versus unadjusted; covariates and model		
Competing-risk analysis; censoring; missing data and attrition		
Gene-specific findings versus overall CH association		
Null/inconsistent findings and sensitivity/subgroup analyses		
Study conclusion and source location		
Outcome domain checklist	t-MN/secondary myeloid malignancy; cytopenia/recovery; transplant outcomes; CRS; ICANS; other CAR-T hematotoxicity; cardiovascular events; survival/mortality; cancer response/control; treatment interruption; other survivorship endpoint.	
Clinical relevance and limits on actionability		
Interpretation guidance	An observational association does not establish causality or benefit from changing effective cancer therapy.	
**F Synthesis mapping**		
1 Definitions and terminology		
2 Therapy-specific selection pressures		
3 High-risk genomic architecture		
4 Longitudinal clonal dynamics		
5 Clinical consequences		
6 Study design and methodological heterogeneity		
**G Uncertainties and verification**		
Main finding in one or two factual sentences		
CH as treatment consequence / outcome modifier / both / unclear		
Confounding: age, cancer, prior therapy, comorbidity, marrow reserve		

Baseline sensitivity, assay changes and variant-origin uncertainty		
Outcome definitions, follow-up and competing-risk limitations		
Pediatric/adult separation; generalizability		
Outstanding information and author-contact need		
Corrections on recheck; amendment date and reason		
Verification status	Not checked / checked by the same extractor / independently checked; specify the actual process.	
Verifier name and date; unresolved discrepancies		
Verification guidance	Record the process actually performed. These fields do not imply independent dual review or a validated risk-of-bias assessment.	
Additional clone/outcome records attached; linked source files		
